# Self-assembled short peptides: Recent advances and strategies for potential pharmaceutical applications

**DOI:** 10.1016/j.mtbio.2023.100644

**Published:** 2023-04-25

**Authors:** Shihua Yang, Mingge Wang, Tianye Wang, Mengchi Sun, Hanwei Huang, Xianbao Shi, Shijie Duan, Ying Wu, Jiaming Zhu, Funan Liu

**Affiliations:** a Department of Surgical Oncology and General Surgery, The First Hospital of China Medical University, Key Laboratory of Precision Diagnosis and Treatment of Gastrointestinal Tumors, China Medical University, Ministry of Education, Shenyang, 110001, China; b School of Pharmacy, Shenyang Pharmaceutical University, Shenyang, 110016, China; c Department of Phase I Clinical Trials Center, The First Hospital of China Medical University, Shenyang, 110102, China; d Department of Anus and Intestine Surgery, The First Hospital of Dalian Medical University, Dalian, 116000, China; e Department of Pharmacy, The First Affiliated Hospital of Jinzhou Medical University, Jinzhou, 121001, China

**Keywords:** Self-assembly, Short peptides, Peptide-based therapeutics, Stabilizers, Imaging agents

## Abstract

Self-assembled short peptides have intrigued scientists due to the convenience of synthesis, good biocompatibility, low toxicity, inherent biodegradability and fast response to change in the physiological environment. Therefore, it is necessary to present a comprehensive summary of the recent advances in the last decade regarding the construction, route of administration and application of self-assembled short peptides based on the knowledge on their unique and specific ability of self-assembly. Herein, we firstly explored the molecular mechanisms of self-assembly of short peptides, such as non-modified amino acids, as well as Fmoc-modified, N-functionalized, and C-functionalized peptides. Next, cell penetration, fusion, and peptide targeting in peptide-based drug delivery were characterized. Then, the common administration routes and the potential pharmaceutical applications (drug delivery, antibacterial activity, stabilizers, imaging agents, and applications in bioengineering) of peptide drugs were respectively summarized. Last but not least, some general conclusions and future perspectives in the relevant fields were briefly listed. Although with certain challenges, great opportunities are offered by self-assembled short peptides to the fascinating area of drug development.

## Graphical abstract

**Figure:**
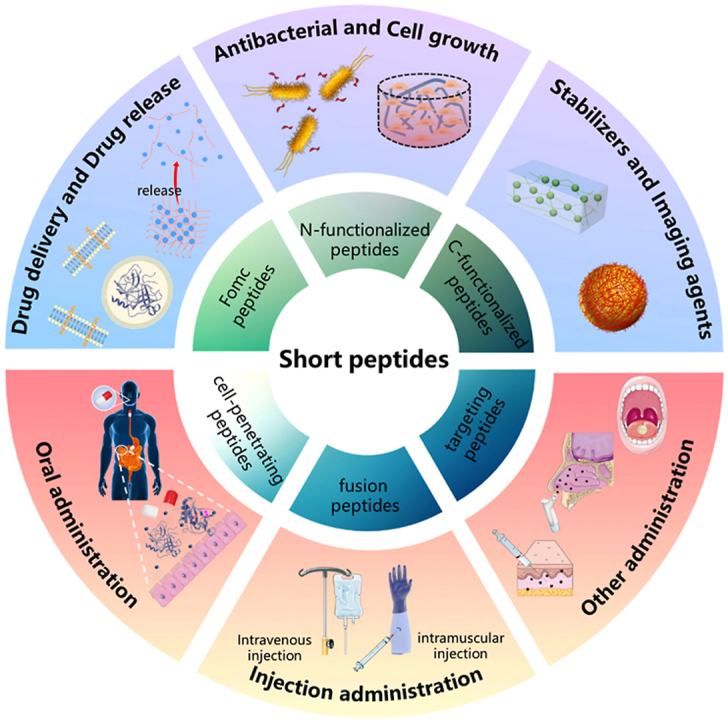

## 1 Introduction

Short peptides have attracted much attention due to the low synthesis costs and convenient modulation compared with other macromolecules [1]. Besides the above properties, self-assembled short peptides are demonstrated good biocompatibility and biodegradability, low immunogenicity and toxicity, and fast response to external stimuli; thus are widely used in drug delivery, vaccination, tissue engineering and cell culture [2,3]. Different self-assembled nanostructures of short peptides are dependent on the inter- and intramolecular forces such as H-bond formation, π−π stacking, and hydrophobic interactions [4]. Moreover, the modified self-assemblies are applied in prodrug strategy to provide unique and specific functionalities and confer desirable pharmaceutical characteristics, via covalent modifying functional moieties or drugs. As is repeatedly reported in current progress, the 9-fluorenylmethyloxycarbonyl (Fmoc) is widely used as the main protective group of the amine in the synthesis of peptides, because their inherent hydrophobicity and aromaticity are key factors to promote self-assembly [5]. However, the self-assembly and applications of modified amino acids and short peptides have not been extensively summarized. Peptides can be divided into cell-penetrating, fusion and targeting peptides according to their way to interaction with biological membrane. These three types of peptides play unique roles in the drug delivery process which can effectively improve the problems of cytotoxicity and low cell specificity of the drug delivery process [6].

Since peptides are easily inactivated by peptidases and rapidly cleared by the kidneys and liver, the half-lives of peptides are often very short. In addition, different routes of administration also affect the half-life parameters of peptide drugs. Various routes of administration have been approved for therapeutic peptide products. Injection is the main route of administration, followed by oral, implanted dosage forms, sublingual and other administration methods [7]. This article will focus on the oral route of administration through gastrointestinal absorption and the parenteral route of administration represented by injection. With the continuous in-depth research on peptide drugs, the diversification and individualization of administration routes will be further developed. Because of their unique structure and stimulation response mechanism, the application of peptides in predicting drug delivery [8] and sustained release has been widely studied [9]. As nano-antibacterial drugs with excellent antibacterial effects, antimicrobial peptides provide a new idea for solving multidrug resistance of microorganisms [10]. Ricardo et al. discussed the emulsification behavior of self-assembled peptides as novel surfactants. Short peptides are structurally diverse and easy to design, compared with traditional surfactants. Self-assembled short peptides have been widely studied as stabilizers of emulsions [11]. Molecular imaging plays an important role in reasonably designing peptide sequences and understanding the supramolecular self-assembly behavior during biological processes [12]. Fluorescence imaging has been widely used to study the dynamic or static behavior of peptides due to its high spatial resolution and small damage. Fmoc, the classical fluorescent structure, plays an important role in characterizing the self-assembly mechanism of short peptides [13]. In addition, composite nanostructures such as supramolecular metallo-hydrogelator and fluorescent groups-based short peptides also have imaging capabilities, showing broad application prospects in biomedicine [14].

This review focuses on several representative constructions, administration routes and applications of self-assembled short peptides in the following order. First, the molecular mechanisms used by different functionalized short peptides that facilitate the formation of self-assembled nanostructures are listed. Next, the routes of peptide administration and the characteristics of cell penetrating, fusion and targeting peptides are summarized. Then, variety of pharmaceutical applications of self-assembled short peptides are discussed, such as stimulus-responsive release, targeted and sustained release, stabilizers, imaging agents, antibacterial agents and in situ self-assembled peptides and injectable hydrogels in bioengineering. Finally, the great challenges and broad prospects of the self-assembled short peptides in the future pharmaceutical area are briefly described.

## 2 Self-assembled peptide designs and hydrogel construction

### 2.1 Short peptides

Proteins and peptides are composed of 20 naturally occurring l-amino acids. All naturally occurring amino acids, except glycine, are chiral and contain carboxylic acids and amino groups. Side-chain groups attached to the chiral carbon are different in each amino acid, forming a diversity of charge, hydrophobicity, size and polarity on the side chain. The number, type and sequence of amino acids can be manipulated to design self-assembled peptides. Peptides can form different structures based on the amino acid sequence [15]. They play an increasingly important role in drug delivery, antitumor and antibacterial drugs, tissue culture, imaging, and membrane protein stabilization (Table 1) [16].

**Table 1:** Typical sequences and structures of peptides.

Short Peptide	Nanostructure	Application	Ref.
Phe-Phe (FF)	Nanotubes	chemotherapy, drug delivery, catalysis,	[17]
I3K	Nanotubes	cell culture，	[18]
Im-KL	Nanotubes	optics, electronics	[19]
L1/HSiW	Nanofibers	antibacterial,	[20]
CFTNF(CPP44-FF-TPP-NH2)	Nanofibers	antitumor,	[21]
Coil29	Nanofibers	immunotherapy, tissue engineering,	[22]
Fmoc-FF	Nanofibrils	drug delivery	[23]
Ac-Ala-Ala-Asn-Cys-Asp (AK)	Vesicle	drug delivery,	[24]
Boc-Phe-Phe-OH	Vesicle	tissue regeneration,	[25]
PRPRPPR	Vesicle	antibacterial	[26]
Fc-FF	Nanobelt	biosensors, chiral optics, thermal quenching	[27]

Short peptides offer various advantages including ease of design, synthesis and characterization, diverse functionalization possibilities, and high biocompatibility and biodegradability. Molecular self-assembly refers to the spontaneous arrangement of individual molecules into ordered structures through non-covalent weak interactions. Self-assembly enables the construction of peptide-based nanomaterials with certain spatial structures. The process of assembly is governed by the balance of attraction/repulsion forces both intra- and intermolecular. The generation of many biological nanostructures occurs through self-assembly processes, such as the formation of the DNA double helix through hydrogen bonding interactions between nucleotide bases, the folding of polypeptide chains to form tertiary or quaternary structures of proteins, and the generation of cell membranes through the self-assembly of phospholipids [28]. Peptides are frequently instable in vivo and are susceptible to enzymatic degradation under physiological conditions. The bioavailability and unfavorable immune response of peptides also limit their further conversion [29,30]. Compared with individual peptides, peptide-based self-assembled nanomaterials with ordered superstructures possess good thermal and mechanical stability and exhibit advantages of semiconductor and optical properties attributed to their structural characteristics [31]. Monomeric polymer molecules generate different hierarchical structures through self-assembly, and play an important role in the fields of chemistry, materials science, and molecular biology [[32], [33], [34]]. The peptides composed of amino acids can self-assemble into different nanostructures, typically with good biocompatibility and bioactivity, promising for a wide range of applications in materials science [35].

Self-assembled short peptides are increasingly investigated as nano-sized materials and hydrogels with a variety of morphologies and characteristics, such as the sphere, cylindricity, and ribbon [36]. Among them, the diphenylalanine (phe-phe, FF) is an elongated tubular assembly discovered in the recognition motif of the Alzheimer's disease β-amyloid peptide [37]. FF is among the smallest self-assembled peptides ever found, as first reported by Reches and Gazit in 2003 [38]. The formation of these smallest self-assembled gels is probably driven by the hydrogen bonds of the dipeptides and π-π stacking interactions between the aromatic residues of dipeptides. The structural characteristics of the amino acids dominate the assembly behavior of most short self-assembled peptides. In general, the fabrication of unique self-assembled peptide nanostructures is mainly based on different apolar, charges, hydrophobic and aromatic side chains of different amino acids [39]. In addition, computational tools effectively screen the self-assembly feature of potential short peptides sequences in the aqueous environment. Frederix et al. rapidly screened 400 dipeptide combinations and predicted their ability to aggregate as a potential self-assembled precursor using coarse-grained molecular dynamics (CG-MD) [40]. Furthermore, they also reported a CG-MD protocol for screening the assembling behavior of 8000 tripeptides. The computational method was verified by analyzing the results of the simulations in terms of aggregation propensity, structural features, and general design rules. The added value of this method in identifying new self-assembled peptides was suggested [41].

Theoretically, the construction of assembled nanostructures may not be static and can be under the dynamic control in different environments. The smart short peptide self-assembly undergoes morphological changes in response to specific external stimuli (pH, temperature, and effect of the enzyme). For example, self-assembled FF forms nanotubes. The morphology and size of the self-assembly are strongly influenced by the pH. Indeed, the increase in pH leads to the increase in the diameter of short tubes, in turn resulting in the formation of hollow tubes [42]. In addition, self-assembled short peptides often reorganize with temperature changes. Cox and colleagues found that the synthetic short peptide gels (Ile-Ile-Ile-Lys, I_3_K) show a hardening pattern mediated by the temperature. The strength of the gels increases by 6-fold when the temperature increases from 20 to 40 ​°C [43]. Another vital factor leading to structural changes is the existence of enzymes. The surfaces of cancer cells always overexpress and secrete endogenous substances such as phosphatases. Kuang and coworkers developed a drug delivery strategy based on phosphatases-catalyzed self-assembled short peptide derivative that selectively accumulates on the surface of cancer cells, finally killing them. Overexpressed phosphatases in cancer cells dephosphorylate the precursors of short peptides to form the self-assemble hydrogels, which are selectively formed around cancer cells, preventing cellular mass exchange to induce the apoptosis of cancer cells [44].

### 2.2 N-functionalized peptides

The addition of various N-terminal capping groups in peptide synthesis has been reported to broaden the structures and functions of the self-assembly short peptides. The aromatic group at the N-terminus facilitates the self-assembly ability via π−π stacking interactions and hydrophobic interactions.

#### 2.2.1 9-Fluorenylmethyloxycarbonyl (fmoc) peptides

Fmoc is widely used as a main amine protecting group to enhance the self-assembly ability of short peptides by hydrophobic and π-π stacking interactions of the fluorenyl rings and hydrogen bonding of the carbonyl group [45]. The hydrophobic groups and π-π stacking confer hydrophobicity and aromaticity of the Fmoc peptides, in which Fmoc is a protective group for amines in peptide synthesis, and the short peptides modified by this group can self-assemble in an easier manner. These modified short peptides self-assemble in a unique kinetic and thermodynamic manner thanks to the additional driving forces provided by Fmoc, such as hydrogen bonding and the interaction of aromatic and hydrophobic bonds in the fluorenyl ring [46].

Fmoc-modified single amino acids are the simplest biomolecules and some of them form a gel by self-assembly. Hydrogels can be prepared from Fmoc-phenylalanine (Fmoc-F) or Fmoc-tyrosine (Fmoc-Y), as revealed by Sutton and coworkers [47]. Their study found that the storage modulus of Fmoc-Y hydrogels is significantly higher than that of Fmoc-F hydrogels, due to the rheological differences in the hydrogels. Fmoc-modified di- and tripeptides have excellent self-assembly potentials for assembling into nanostructures. Fmoc-FF is the most widely studied Fmoc-modified dipeptide [48]. Mechanistically, Fmoc-FF can self-assemble into nanowires, resulting in rigid hydrogels. The addition of sodium alginate can change the movement and combination capacity of Fmoc-FF molecules during the assembly process, finally regulating the morphology of the hydrogels [49]. This self-assembly forms a 3-dimensional fibril network with pores from 10 ​nm to 1 ​μm. The fibrils of the peptide membrane serve as a stabilizer, preventing the disintegration at the floating interphase.

**Fig. 1:**
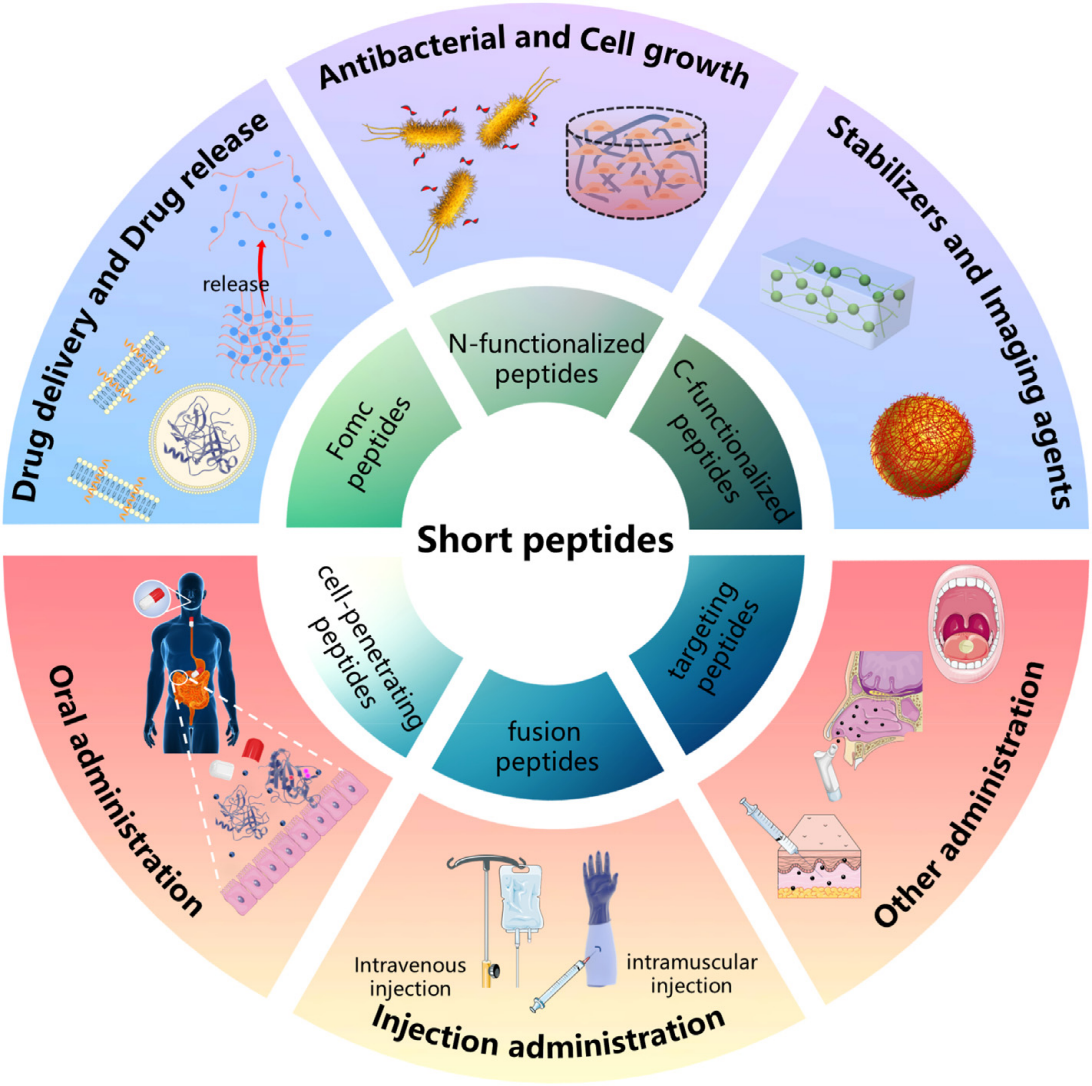
Schematic illustration of short peptide-based self-assemble nanostructures and applications.

The final pH of the Fmoc-FF gels is the key determinant of the mechanical properties, and experimental factors, such as the fraction of DMSO (dimethyl sulfoxide) or the nature of the buffers, generate additional variability. Smith and coworkers discovered that the molecular assembly of Fmoc-FF is an anti-parallel β-sheet structure. Four twisted anti-parallel β-sheets interlock through π−π stacking interactions and form a cylindrical structure. These cylinders line up side-by-side to create a flat ribbon [50]. The electrostatic interactions also play an important role in controlling the self-assembly of Fmoc-modified bio-blocks and not only hydrophobic interactions and π−π stacking. Xie et al. designed a simple Fmoc-FWK tripeptide derivative sensible to the pH change, and capable of forming different assemblies, such as nanofibers, large and flat ribbons, as well as helical nanoribbons [51]. The Fmoc-FWK tripeptide can self-assemble into nanofibers at pH 5.0 due to the electrostatic interactions between the charged carboxyl groups and the amino groups on the K residues. When the pH increases to 6–11, it turns into larger flat ribbons composed of many nanofibers. Then, it twists into a left-handed helix at pH 11.2–11.8, finally returning to nanofibers at a very basic pH of 12.0. Morphology change is ascribed to electrostatic interactions, π−π stacking, and hydrogen bonding produced by the ionization of carboxyl and amino groups of short peptides (see Fig. 1).

**Fig. 2:**
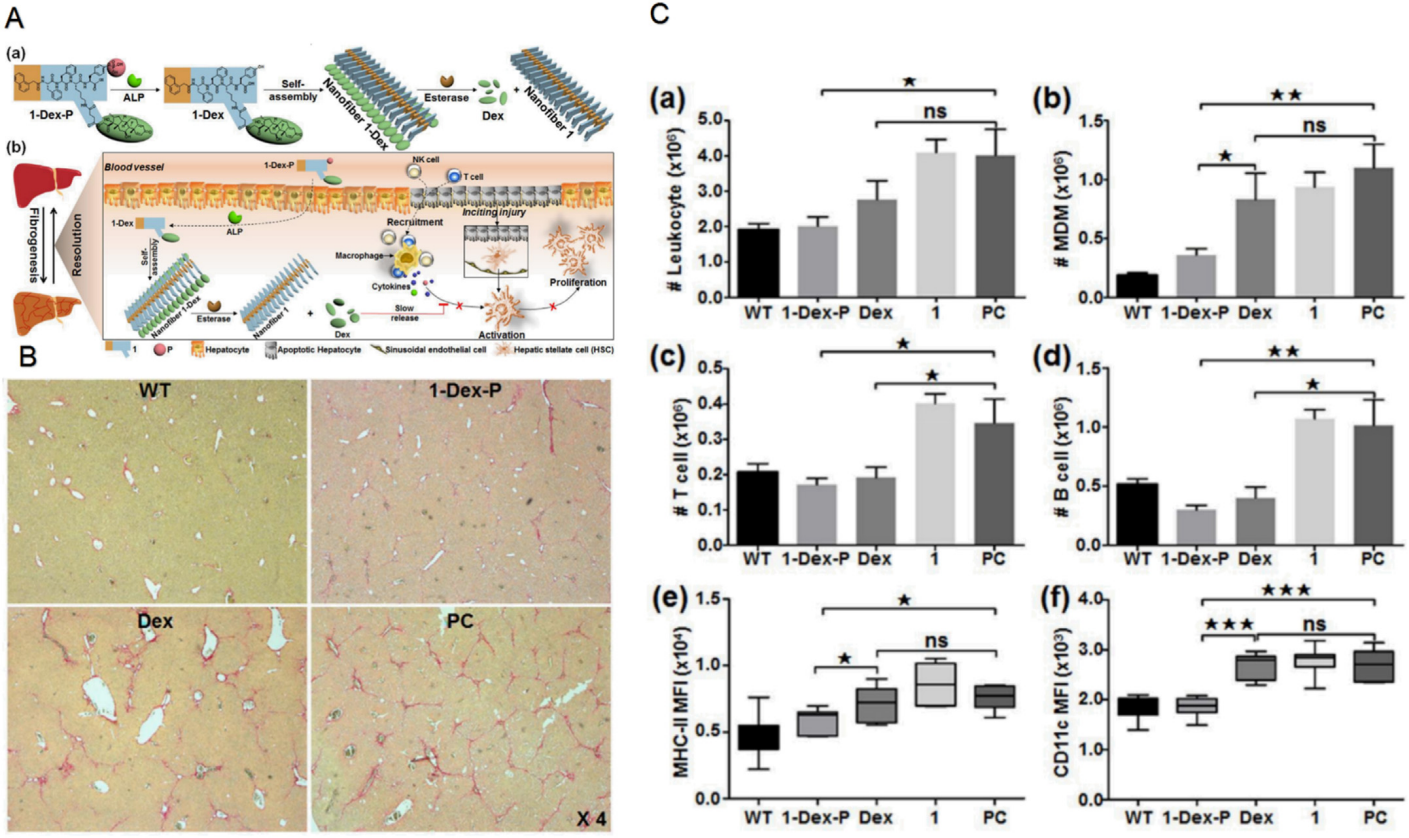
**A**. (a) The tandem ALP-instructed self-assembly of 1-Dex-P to form nanofiber 1-Dex, and esterase-controlled slow release of Dex from the nanofiber. (b) Schematic illustration of tandem enzymatic strategy in hepatic fibrogenic parenchyma. **B**. Representative Sirius Red staining images of the liver slices of the mice in different groups at 72 ​h after the last injection. **C**. Summary bar graphs of the liver CD45^+^ total leukocyte (a), CD45^+^ CD11b ​+ ​F4/80low monocyte-derived macrophage (MDM) (b), CD45^+^ CD3^+^ NK 1.1-T cell (c), and CD45^+^ CD19^+^ B cell (d) calculated from CCl4-induced hepatic fibrosis mice sacrificed after different treatments. Summary graphs of the relative amounts of inflammatory molecules MHC-II (e) and CD11c (f) on liver resident Kupffer cell. Adapted with permission from Ref.32 Copyright 2018, American Chemical Society. (For interpretation of the references to colour in this figure legend, the reader is referred to the Web version of this article.)

The enzyme-triggered self-assembly strategy has recently caused wide scientific concern. Enzyme-instructed self-assembly (EISA) and co-assembly strategy were proposed by Tang et al. and demonstrated to significantly improve the anti-inflammatory ability of dexmedetomidine [52]. A tandem enzymatic self-assembly and slow-release strategy was developed to design the hydrogelator precursor Nap-Phe-Phe-Lys (Dex)-Tyr (H_2_PO_3_)–OH (1-Dex-P) based on the EISA strategy. Dexmedetomidine is self-assembled at the site of liver fibrosis and exhibits good anti-fibrotic properties (Fig. 2) [53]. The dexmedetomidine hydrogel precursor of nap-phenylalanine (Dex)-Tyr (H_2_PO_3_)–OH (1-Dex-P) is first dephosphorylated by alkaline phosphatase (ALP) to obtain the hydrogel nap-phenylalanine (Dex)-Tyr-OH (1-Dex), which self-assembles to form nanofiber 1-Dex. Then under esterase hydrolysis, the morphology of nanofiber changes and Dex is slowly released. The ester bond of the 1-Dex side chain is hydrolyzed to convert into nanofibers with different spatial configuration and slowly releases dexmedetomidine, which in turn inhibits the activation and propagation of hematopoietic stem cells by suppressing the stimulatory effect of their upstream infiltrated inflammatory cells and the pro-inflammatory effect of Kupffer cells [54]. Moreover, the release of dexmedetomidine from its parental prodrug 1-Dex-p is esterase dose-dependent. The properties of supramolecules make hydrogels respond to external stimuli such as pH, ionic strength, and temperature change，thus exhibit the superior characteristics of high selectivity. Besides, with mild reaction conditions as another prominent advantage of biocatalysis, the synthesis of self-assembled materials facilitated by biocatalysts has become an area of interest. In one of the studies, two lipases including the yeast C. rugosa lipase (CRL) and the bacterial *P. cepacia* lipase (PCL) were respectively tested for the ability to catalyze the synthesis of Fmoc-modified tripeptide (Phe3) from Fmoc-phenylalanine (Phe) and dipeptide FF (Phe2). As a result, PCL was shown to trigger the self-assembly of peptide hydrogels by reverse hydrolysis. Therefore, the ideas for the application of hydrogels in tissue engineering is provided [55].

#### 2.2.2 Other N-functionalized peptides

Naphthalene (Nap) is preferred to Fmoc in some cases, and especially the Nap-containing hydrogels have been extensively studied. Laverty and coworkers synthesized a group of Nap-peptides molecules. These molecules spontaneously assemble into an antimicrobial hydrogel at physiological pH (pH 7.4). The 2% w/v Nap-phenylalanine-phenylalanine-lysine-lysine (Nap-FFKK) hydrogels possess the powerful antibacterial activity, significantly reducing the biofilm of viable *Staphylococcus epidermidis* by at most 94% [56]. The Nap-based peptide amphiphile hydrogelators are considered as an ideal candidate for stabilizing the protein in the 3-D dimensional matrix and reducing the degradation of proteins by the protease. Wang et al. were the first to report the use of Nap-glycine-phenylalanine-phenylalanine (Nap-GFF) hydrogels to avoid the degradation of complex proteins by a simple encapsulation strategy. This method maintains the activity of the encapsulated protein, stabilizes the encapsulated protein at room temperature, and improves the mechanical property of the protein [57].

The aromatic group Ferrocene (Fc) has attracted considerable attention due to the rational and precise design of chiral nanostructures. The hierarchical self-assembly of a Fc-modified dipeptide such as Fc-phenylalanine-phenylalanine (Fc-FF), changes the conformation of the secondary structures from flat β sheets into twisted β sheets with the addition of counterions [58]. In addition, pyrene has been studied in short peptide modification fields. The pyrene-based peptide amphiphile hydrogelators increase ∼10^6^-fold the storage modulus compared with FF alone [59]. Ambidextrous gelators, simultaneously acting as both low-molecular-weight hydrogelators and low-molecular-weight organogelators, are rarely reported. Mandal and coworkers designed short peptides consisting of three pyrene-containing amino acid derivatives that can efficiently gelate under both organic and aqueous solvents. The organogelation efficiencies of the three gelators are in the range 0.7–1.1% w/v in various organic solvents, while their hydrogelation efficiencies are 0.5–5% w/v in aqueous solvents at acidic pH values (pH 2.0–4.0) [60]. Azobenzene is the most widely used type of photoswitch for the photo-control of biomolecules. The addition of an aromatic group can potentially enhance the aromatic stacking interactions within the nanostructure, strengthen the stability of hydrogels systems, and obtain materials with a certain number of properties. The aromatic group can facilitate the self-assembly of short peptides, simultaneously determine the role of functionalization of the self-assembled peptide. For example, photo-responsive hydrogels have received much attention in materials science because their characteristics and functions are subjected to the temporal and spatial regulation of the 3D culture microenvironment dynamically controlled by light irradiation [61]. Kim et al. reported cross-link self-assembled β-sheet nanofibers formed by R-lysine-lysine(R)-NH_2_ dipeptides, in which R is composed of 7-DAC (7-(diethylamino)-3-coumarin carboxylic acid). Under the irradiation of 365 ​nm, the coumarin moieties crosslink, while the nanofiber structure retains in 2,2,2-trifluoroethanol (TFE), which is a potentially denaturing solvent for dissolving the non-crosslinked material [62]. Moreover, aromatic heterocycles can be modified at the N-terminal end and perform certain functions. The indole capping groups are reported to attach to the short peptides GFFY and GDFDFDY and construct short peptide-based hydrogenation reactors. With the moderately strong mechanical properties, short peptide-based supramolecular hydrogels Indol-GFFY and Indol-GDFDFDY form flexible and uniform nanofibers that intertwine to form a three-dimensional network for hydrogenation in aqueous solution. According to further cytotoxicity assays, the hydrogels are demonstrated to have high biocompatibility and good potential as vaccine adjuvants [63]. Based on a nonspecific endoprotease (the thermolysin from Bacillus thermoproteus rokko), NDI-YF-NH2 is formed by enzymatic condensation of NDI-functionalized tyrosine (NDI-Y) and phenylalanine amide (F–NH2), and thermodynamically driven to produce nanostructures with optimized supramolecular interactions. In 100 ​mm phosphate buffer (pH 8), the precursor NDI-Y (10 ​mm) alone forms a yellow-orange self-supporting hydrogel within 2 ​min and is stable for several months [64]. NI is reported to be used as a cap in a neutral environment to bind GFF to form a fluorescent hydrogel, which is transparent with nickel as the chromophore and formed by the introduction of the NI group at the N-terminal end of the tripeptide NI-GFF (NI-Gly-LPhe-L-Phe). NI-GFF is successfully hydrogenated at pH 7.0 to present a homogeneous fibrous network with a diameter of 11.02 ​± ​2.0 ​nm as observed by TEM images. In addition, NI-GFF can form self-assembled aggregates under neutral conditions, which implies a reduced cytotoxicity. Under physiological conditions, NI-GFF also exhibit good biostability illustrated by good resistance to enzymatic digestion while incubating with proteinase K. Therefore, NI-GFF is a candidate material for tissue engineering and drug delivery applications [65].

It is well known that some diseased cells like cancer cells are prone to the production of reactive oxygen species, thus self-assembled peptides based on the redox process have been investigated. BPmoc-FF, BPmoc-FFI, BPmoc-FFL and BPmoc-FFF, a series of benzyl-capped tripeptides substituted with boronic acid, were reported by Ikeda et al. [66]. Based on the facts that FFF is extremely sensitive to hydrogen peroxide and its hydrogel network can be completely disintegrated by hydrogen peroxide, the multi-component hydrogel containing the synthetic amplifier, sarcosine oxidase and urate oxidase was utilized to create a naked eye detection sensor for the level of plasma uric acid [67]. NpxFFKK with a critical micelle concentration of 0.4% w/v exhibits the greatest ability to form stable hydrogels compared with 2% w/v IbuFFKK and 1.5% w/v IndFFKK. The antibacterial effects of the four hydrogels were separately measured against the methicillin-resistant *Staphylococcus epidermidis* (ATCC 25984), *Staphylococcus aureus* (ATCC 6584), *Pseudomonas aeruginosa* (PAO1) and *Escherichia coli* (ATCC 11303), and NpxFFKK is the only NSAID-peptide with significant concentration-dependent bactericidal effects against all the four isolates. Specifically, NpxFFKK at 1.5% w/v and above, 1.0% and above, and 0.5% w/v and above, respectively, shows significant bactericidal effects on the Gram-negative *Pseudomonas aeruginosa* and *Escherichia coli*, the Gram-positive *Staphylococcus epidermidis*, and the *Staphylococcus aureus*. The antibacterial effect of NpxFFKK is significantly higher than that of NSAID due to the combined effect of the local cationic charge on the hydrogel surface and the porous network of the cross-linked nanofibers [68].

### 2.3 C-functionalized peptides

Hydrogels with C-terminal modification can be more stable than those with unprotected C-terminus, thus the hydrogels formed from C-functionalized peptides are desired. As regards Fmoc-FF, the ionization of the unmodified peptide C-terminus is of crucial importance for the self-assembly property of hydrogels. The ionization of the α-carboxylic acid groups of the amino acids is dependent on the pH of the medium. Tang and coworkers investigated in detail the effect of pH on the self-assembly process of Fmoc-FF into fibrils. The pK_a_ of Fmoc-FF molecules is approximately 6.4 and 2.2. Most ionized molecules are soluble at high pH and no self-assembly occurs. When the pH value reaches nearby pK_a1_, the ionized and non-ionized molecules self-assemble into pair fibrils which are the antiparallel β-sheet structures and negatively charged. Once the critical gelation concentration is reached, the fibrils entangle, and the three-dimensional-network hydrogels are formed. When the pH is equal to ∼pK_a2_, the neutralization of most peptides leads to the aggregation, then precipitation and last phase separation of the hydrogels [50]. In addition, a family of tetrapeptides with alkyl groups as the C-terminal protecting groups is used to investigate the roles of protecting groups in hydrophobic/hydrophilic balance and peptide fibrosis, and the interaction potentials of family compounds with amyloid Aβ1-40. The family of isomeric tetrapeptides contain both non-polar aromatic phenylalanine (F) and polar aspartic acid (D) residues. Under the influence of pH, various molecular hydrogels can be formed based on different binding abilities of different tetrapeptide family compounds, and hydrophobic and π-stacking interactions are important for the hydrogel formation. Furthermore, the amino acid sequences are the deciding factors for the interactions between the tetrapeptide family compounds and Aβ1-4 [69].

The solubility is a huge challenge for peptide-based nanostructures because the self-assembled peptides consist of hydrophobic moieties. The saccharide modifications on the C-side solve this problem quite effectively. Two nanovectors for drug delivery were designed by attaching the C-terminus of Boc–FF–Ahx-GA and H-FF-Ahx-GA with glucosamine (GA) via a 6-aminocaproic acid (Ahx) linker, and presented a good illustration for the formation of nanostructures in aqueous media [70]. Xu reported a biocompatible glycopeptide incorporating a Fmoc-FFD sequence and a therapeutic glucosamine moiety. This glycopeptide can build a self-assembled hydrogel through hydrogen bonds and π−π stacking. The therapeutic glycopeptide hydrogels have a great potential in the treatment of slight diseases due to the convenience of treatment and the absence of toxicity [71]. The self-assembled Fmoc-F5-Phe-OH was demonstrated to rapidly form the hydrogels, while Fmoc-F5-Phe–OH–derived fibrils showed unstable low water solubility [72]. The novel sequence Fmoc-F5-Phe-PEG was designed to solve this problem. The addition of polyethyleneglycol (PEG) chains increases the solution phase stability of fibrils with an ideal stress-responsive behavior [73]. The PEGylated long-chain peptide analog has a slightly lower biologic activity than the unmodified analog *in vitro*, but has a significantly prolonged action time and a high quality pharmacodynamics in vivo. Therefore, PEGylation strategy is widely used in protein-based medicine, the details of which are beyond our scope and not discussed here [74]. The above evidence demonstrates that C-terminal modifications have a great effect on the solubility and stability of self-assembled materials.

The hydrophilic amino acid chirality at the C-terminus plays a key role in determining the chirality of self-assembled nanostructures formed by short amphiphilic peptides [75]. Accordingly, three enantiomeric isomers, such as ^L^I_3_^L^K and ^D^I_3_^D^K, ^L^I_3_^D^K and ^D^I_3_^L^K, and ^La^I_3_^L^K and ^Da^I_3_^D^K, were designed [76]. The torsional chirality of the supramolecular nanofibers is determined by the chirality of the hydrophilic Lys head at the C-terminus. As revealed by TEM, AFM, and SEM, the ^D^I_3_^L^K nanofibers have the same left-handed twist as ^L^I_3_^L^K when the three hydrophobic L-Ile residues are substituted by three D-Ile residues. Similarly, the ^D^I_3_^L^K nanofibers showed the same right-handed twist as ^L^I_3_^D^K when the hydrophilic L-Lys residues are replaced by their enantiomers (D-Lys).

The aromatic-aromatic interactions with the involvement of the C-terminus can transform the secondary structure of the self-assembled peptides. Based on C terminal modifications of aromatic motifs and/or pyrene, two complementary pentapeptides, while mixed together, were found to change structures from α-helical as the single compound to β-fold via the aromatic-aromatic interaction [77]. In the experiment, the decapeptide Arg-Met-Leu-Arg-Phe-Ile-Gln-Glu-Val-Asn (RMLRFIQEVN) was divided into two pentapeptides RMLRF (A) and IQEVN (B), with pyrene linked at the C-terminus, preparing for the aromatic ring interactions. As shown by CD spectra, A-Py and B-Py molecules themselves tend to be α-helices, but form β-sheet structures while mixed. As the concentration of the A-Py and B-Py mixtures decreases from 2.5 ​mM to 0.040 ​mM, the peaks nearby 375 ​nM and 400 ​nM increase. When the concentration reaches 0.04 ​mM, the ratio of the peak intensity at 470 and 375 is nearly zero, and α-helical structure is increasingly observed in solution. The experiment suggests that aromatic-aromatic interactions promote the secondary structure transformation of pentapeptides.

Peptide self-assembly has created a vast array of peptide-based materials tailored for specific applications, range from three-dimensional cell culture scaffolds to immunotherapeutic, antibacterial and anticancer drugs. Self-assembly processes are mediated by non-covalent interactions, including van der Waals, electrostatic, hydrogen bonding and stacking interactions. Self-assembly of peptides is a hierarchical process, and self-assembled peptides can generally be classified into two categories, α-helix and β-sheet (including β-hairpin), based on the secondary structure formed. As component units of peptides, amino acids have different physicochemical properties. Due to the diversity of charges, hydrophobicity, size and polarity of peptides on the side chains, both their number, type and sequence affect the process of self-assembly. Peptides can form different structures based on the amino acid sequence [16]. Although side-chain interactions of peptides are generally considered to be critical in the self-assembly process, the process of how they determine secondary structure to achieve higher scale and complexity of structures is in urgent need of more in-depth exploration in the field of achieving controllable fabrication of nanostructures and nanomaterials [78]. Xu et al. designed a series of Ac–I_3_XGK–NH_2_–based peptides, demonstrating the ability and potential of designing and fabricating unique nanostructures and nanomaterials by controlling structural and interaction processes in future [79]. In order to reveal more clearly the intermolecular forces during the self-assembly of short peptides and N/C-functionalized short peptides, we compiled the molecular structures of relevant short peptides mentioned in the paper and their corresponding self-assembly forces (Table 2).

**Table 2:** Molecular structure of peptides and the non-covalent forces involved in self-assembly process.

Peptides			Molecular structure	Self-assembly drivers	Refs
Short peptide		FF		hydrogen bonding,	[80]
				π−π stacking	
		I3K		hydrogen bonding,	[81]
				hydrophobic interaction	
N-functionalized peptide	Fmoc peptide	Fmoc-FF		hydrophobic interaction,	[50]
				π−π stacking	
		Fmoc-FWK		electrostatic interactions,	[50]
				π−π stacking,	
				hydrogen bonding	
		Fmoc-FFF		electrostaticaromatic interactions,	[51]
				hydrogen bonding	
	Other N-functionalized peptide	Nap-FFKK		π−π stacking,	[56]
				hydrogen bonding,	
				van der waal's interaction	
		Fc-FF		hydrogen bonding,	[78]
				π−π stacking,	
				electrostatic interactions	
		NI-GFF		π−π stacking,	[82]
				hydrogen bonding	
C-functionalized peptide		ZFDFD/		hydrophobic interactions,	[83]
		ZDFDF/		π−π stacking,	
		ZFFDD/		hydrogen bonding	
		ZFDDF/			
		ZDFFD			
		H-Phe-ΔPhe-εAhx-GA		hydrophobic interactions	[84]
		Fmoc-F5-Phe-PEG		π−π stacking	[73]

As we discussed above, short peptides can be classified as CPP, targeting peptide, and fusion peptide based on the way they interact with biological membranes, which are functionally classified from a biological point of view. The N/C-functionalized short self-assembling peptides, on the other hand, are classified chemically based on the structural characteristics of the constituent units. The structural features of peptides usually determine their functionality, and peptides exhibit functions and characteristics that are externalizations of their structure and composition. CPPs have extensive physicochemical properties and different structures which are endogenous and/or similar to endogenous proteins with good biocompatibility and non-immunogenicity [85]. Based on their physicochemical properties, CPPs can be classified as: (i) cationic peptides containing the presence of arginine and lysine residues (ii) hydrophobic peptides containing a large number of hydrophobic amino acids such as alanine, methionine or valine (iii) amphiphilic peptides containing amphiphilic amino acid amino acids. Thus, changing the amino acid composition of a CPP affects its charge and hydrophilic/hydrophobic properties. Furthermore, modification of the CPP backbone/amino acid residues can improve the stability of the CPP [86]. Rennert et al. modified the human calcitonin (hCT)-derived penetrating peptide and found that it prevents protein degradation when d-phenylalanine and/or N-methylphenylalanine residues are substituted [87]. CPPs can re-sensitize drug-resistant cells in combination with antineoplastic drugs [88]. CPPs also possess the property of self-assembling short peptides, which are prepared as pre-drugs by coupling with drugs. Specific environments (pH, enzymes, light and heat radiation) drive self-assembly or structural changes. Resulting in the release of the drug and facilitating cellular uptake [89]. CPPs present superior spatial entry capability capacity. However, the delivery efficiency of single peptides is often limited, and a number of peptides require additional coupling reactions. How to design a simple and useful vehicle requires an efficient delivery vehicle with no additional reactions. Hwang et al. reported a novel class of fusion peptides and investigated their potential as siRNA vectors [90]. The three fusion peptides in the study are linked by SPACE, cationic oligoarginine (R7, R11 and R15) and GCG sequences. The fusion peptides successfully form stable self-assembled nanocomplexes with siRNA and exhibit enhanced cellular uptake of sirna via electrostatic attraction. The design of tumor-specific targeting receptors is critical in tumor-targeted delivery. An important factor for effective tumor-targeted delivery is the cancer-specific de novo- or over-expression of target receptors, and their properties on the tumor cell surface [91]. Peptides can be designed to engage these receptors acting as tumor-targeting ligands [92]. Chemical modification of peptides can effectively improve the stability of peptides [93]. Jing et al. reported that a peptide (RS) evolved from the hepatocellular carcinoma (HCC) targeting peptide P47 that exhibits higher tumor tissue targeting. The peptide induces nucleolar stress when combined with the chemotherapeutic agent oxaliplatin (OXA) (RS-OXA), showing both cellular and subcellular targeting. The study showed that RS is able to localize to the nucleolus, enabling functional imaging and targeting delivery of OXA in HCC mice, and providing a versatile tool for tumor imaging and targeted therapy [94].

Some short peptides exhibit cell-penetrating, fusion, and targeting characteristics and function by self-assembly, covalent linkage, or co-delivery. CPP, targeting peptide, and fusion peptide are peptides with various structures and compositions, which could be either short peptides or more complex peptides.

Peptides are ideal biomaterials for their unique bioactivity, biodegradability and biocompatibility. Peptides are usually modified by alkylation and acetylation to increase their stability [95]. By modulating non-covalent interactions, peptides are able to self-assemble into dynamic nanostructures [96]. The peptide has a distinct secondary structure (α-helix, β-sheet, etc.). Hydrogen bonds between the main chain amides induce the formation of α-helix structures. π-π interactions are one of the important interactions in building peptide-based nanomaterials. A series of aromatic-based chemically coupled peptide derivatives (Naphthalene, Fmoc, Bispyrene (BP), etc.) were developed to construct self-assembled nanomaterials with π-π interactions [97].

Peptide amphiphiles (PAs) consist of hydrophobic ends (hydrophobic alkyl groups) and hydrophilic ends (peptides). When PAs linked with alkyl chains are exposed to aqueous solutions, the hydrophobic tail of the peptide can induce and/or stabilize the three-dimensional structure of the peptide head group, driving the self-assembly process. The sequence of β-shees formed by PAs, usually with diameters between 6 and 10 ​nm and lengths up to several micrometers, self-assembles into cylindrical nanofibers (NFs). When the structural elements of amphiphilic molecules are modified, the morphology, function and surface characteristics of the variable PAs are changed in certain ways [98].

Therefore, the physicochemical parameters of nanomaterials are essential for imaging and drug delivery with respect to effectiveness during circulation, penetration, and cellular uptake. For example, small nanoparticles exhibit deep penetration characteristics [99]. However, large nanoparticles usually have a long circulation in the blood, enabling long-term imaging and high accumulation of drugs [100]. Nanoparticles with negatively charged surfaces can be retained for longer periods of time during blood circulation. Cell membranes often exhibit a negative charge, thus nanoparticles with positively charged surfaces can be more readily taken up by cells, however, nanoparticles with positively charged surfaces are rapidly cleared after non-specific uptake by cells, triggering serum inhibition [101].

Self-assembled nanomaterials which have intrinsic instability may undergo structural, morphological and functional changes at the biological interface. In the face of these challenges, more stable self-assembled nanomaterials need to be developed to cope with the complex and variable biological environment. Meanwhile, there is also a need to fully exploit the advantages of their dynamic nature to construct in situ nanomaterials under the stimulation of specific biological conditions for the signal conversion with a view to further developing self-assembled peptide-based nanomaterials for life science and biology applications such as the treatment and diagnosis of diseases.

## 3 Route of administration

### 3.1 Peptide-based drug delivery

Chemotherapy is the most widely used cancer treatment modality. However, it often leads to substantial side effects due to the low bioavailability and poor targeting of drugs, frequently requiring high-dose drug treatment. Peptide drug delivery systems show great potential in cancer treatment because of their good targeting ability and biocompatibility, low immunogenicity, and low toxicity [102]. However, nano-drug delivery systems, such as liposomes and polymer nanoparticles, suffer from particle aggregation, cytotoxicity, low drug loading, and low cell specificity. The aforementioned problems can be substantially overcome by combining peptides with nanoparticle delivery systems [103]. Peptides can be classified into three categories according to their biochemical properties, namely cell-penetrating, fusion, and targeting peptides. The successful internalization of drugs at effective therapeutic concentrations is a major hurdle that must be overcome during drug delivery. Cell-penetrating peptides (CPPs) increase cellular uptake of nanoparticles, stimulating cellular internalization (e.g. pinocytosis and clathrin-mediated endocytosis) by interacting with the cell membrane [104]. However, the lack of endocytic escape makes nanocarrier-mediated drug delivery susceptible to lysosomal degradation. Fusion peptides release cargo through fusion reactions, thus enabling endocytic escape [105]. Further, these peptides can enhance membrane affinity and increase the effective release of therapeutic drugs after entering the cells. When fusion peptides were encapsulated or coupled to the nanoparticle system, the system exhibited enhanced cellular uptake and endocytic activity [106]. Targeted drug delivery systems significantly enhance cellular uptake, reduce off-target adverse effects, and improve clinical translatability. Targeting peptides are associated with advantages such as lower manufacturing costs, improved efficiency of infiltrating tumor masses, high stability, and tunability. Targeting peptides increase the specificity of drug delivery systems, targeting tumors and cancer cells, enhancing cancer cell uptake of therapeutic drugs, and minimizing off-target effects [107]. However, the insufficiency of these peptides remains. Non-specific targeting of healthy cells by targeting peptides continues, especially when normal cells also express the target, which is more prevalent. In tumor treatment, continuous deepening of the research on the target region will facilitate the further development of drug-targeted delivery [108].

### 3.2 Route of administration of peptide drugs

Because of the presence of peptidases and the rapid clearance of the kidney and liver, peptides often have a very short half-life in the human body. Moreover, the low cell membrane permeability of peptides makes targeting intracellular receptors and physiological barriers in absorption regions, such as the small intestine, difficult for most low-lipophilic peptides [109].

Various routes of administration have currently been approved for therapeutic peptide products. Among them, subcutaneous is the most common route, followed by intravenous and intramuscular. In addition, delivery strategies such as oral, implanted dosage forms, and sublingual have been developed to some extent [7].

#### 3.2.1 Route of administration: injection

Among parenteral routes of administration, intravenous injection is the main route. It mainly includes subcutaneous injection, intravenous injection, and intramuscular injection. The intravenous route prevents the problems of low drug bioavailability and effectiveness due to drastic pH changes and the complex enzyme environment present in the gastrointestinal (GI) tract.

However, due to the short half-life of peptide drugs, multiple drug injections are required in a short period to maintain a certain blood concentration. This treatment induces constant distress among patients, with poor patient compliance. How to prolong the half-life and reduce the number of injections have become a crucial research topic in injection administration [110].

Radiotherapy has a critical role in the prevention and treatment of tumor development and recurrence. In brachytherapy, short-range radiation sources directly and precisely reach the tumor lesion or the postoperative cavity [111]. Thermosensitive hydrogels have low viscosity and can be easily injected at low temperatures. When the temperature reaches body temperature, the hydrogels become solid-like or highly viscoelastic, exhibiting the potential to deliver radionuclides [112]. The SmacN7 peptide can activate the tumor cell death pathway, thereby mediating the effect of radiation-induced cytotoxicity. Liu et al. developed an injectable supramolecular hydrogel (PETyr) for co-delivery of iodine-131 (I131) and the SmacN7 peptide [113]. They used I131 as the radiation source for brachytherapy. Cell-penetrating peptide-modified SmacN7 peptides (SmacN7-R9 peptides) act as a radiosensitizer, promoting the activation of tumor cell apoptosis pathways and enhancing radiation-induced cytotoxicity. The local retention and the degree of intratumoral infiltration of SmacN7-R9 peptides loaded in the PETyr hydrogel were assessed by detecting the fluorescent signal. The SmacN7-R9 peptides were immobilized in situ and exhibited a higher degree of intratumoral infiltration compared with the SmacN7 peptides. In situ administration can place the thermosensitive supramolecular hydrogel (PETyr-I131) closer to the primary tumor lesion or postoperative cavity, which is beneficial for delivering 3D conformally immobilized radionuclides and radiosensitizers. This administration form can reduce radiation-related side effects while displaying a good synergistic effect, which provides a novel idea for the synergistic treatment of radiotherapy and chemotherapy.

#### 3.2.2 Route of administration: oral

Oral administration is often the preferred route of administration because of its convenience and good patient compliance. However, the low in vivo bioavailability of oral peptide products due to their pH sensitivity, easy enzymatic hydrolysis, and difficulty in crossing the epithelial barrier have hindered their development and commercialization [114]. Peptide drugs exhibit poor chemical and physical stability to external factors. Poor absorption of oral peptide drugs into the blood circulation limits their further clinical applications [115]. During oral administration, peptide drugs face many harsh environments and obstacles after entering the GI tract, which mainly include the superacid pH value in the stomach, enzymatic degradation, and intestinal mucus layer and epithelial layer [116]. Therefore, the bioavailability of oral peptide drugs in vivo is often low. Studies have shown that the bioavailability of orally administered desmopressin acetate, a synthetic analog of the hormone arginine vasopressin used in nocturia treatment [117], is greatly reduced compared with that of the intravenously administered drug.

Structural modifications such as pre-modification, cyclization, and terminal modification can improve the performance of oral peptides [118]. In addition, co-administration strategy is a hot research direction for oral peptides.^26^ When oral peptides are co-administered with enzyme inhibitors, these inhibitors specifically inactivate peptidases, thus increasing peptide bioavailability [119]. Peptide bioavailability increases when oral peptides are co-administered with absorption enhancers, such as CPPs and polymers, which can increase the transport efficiency of peptides by enhancing their permeability across intestinal epithelial cells [120].

Different carrier systems have also been employed to improve the bioavailability of oral peptides. Carriers such as hydrogels and various particle delivery systems improve drug utilization by protecting peptides from enzymatic degradation or increasing their absorption [121]. The huge gastrointestinal mucus layer secreted by goblet cells is a major barrier for oral peptide nanomedicines to enter the GI tract [122]. The mucus layer is composed of cell-associated mucins and glycoproteins, covering the absorptive intestinal epithelium, and is approximately 37–170-μm thick. This layer can trap and rapidly remove larger molecules and pathogens, especially those with cationic properties. Therefore, the ability of oral peptide nanomedicines to penetrate the mucus layer determines the degree of its entry into the intestinal epithelial cell layer. Positively charged particles or those with hydrophobic surfaces have difficulty penetrating the mucus layer. Mucus penetrating particles (MPPs) with a hydrophilic neutral surface can effectively overcome this barrier, especially when modified with polyethylene glycol, which enables the rapid diffusion of MPPs through the mucus layer [123]. Oral peptide drugs with diameters of up to 200 ​nm are more likely to diffuse through the mucus layer since nanoparticles in the GI tract are mainly transported through low-viscosity pores within the elastic in mucus.

To adapt to the complex microenvironment of the GI tract, a series of composite functional materials are used for prescription design, which ultimately leads to an overly complicated drug design. This makes translation of most oral peptide nanomedicine formulations into clinical practice difficult [124]. Some evidence suggests that nanoparticles generally accumulate and stay within the reticuloendothelial system organs of the liver and spleen, whereas <0.7% of the total amount of nanomedicines accumulate at tumor sites. This phenomenon reduces bioavailability and increases drug toxicity to certain organs [125]. Nanomedicines as foreign particles face some biological barriers during pulmonary delivery. (1) The mucociliary escalator in the airway tract removes foreign particles from the lungs. (2) Alveolar macrophages remove foreign particles through endocytosis. (3) The mucus layer on top of the airway epithelium is a strong barrier that captures nanomedicines and prevents them from reaching target cells.

#### 3.2.3 Other routes of administration

In addition to injection and GI administration, other modes of administration can exert unique effects on different diseases. As researchers continue to conduct in-depth research on the route of administration, an increasing number of drugs are administered through new routes to develop more effective and novel therapeutic effects in disease treatment.

**Fig. 3:**
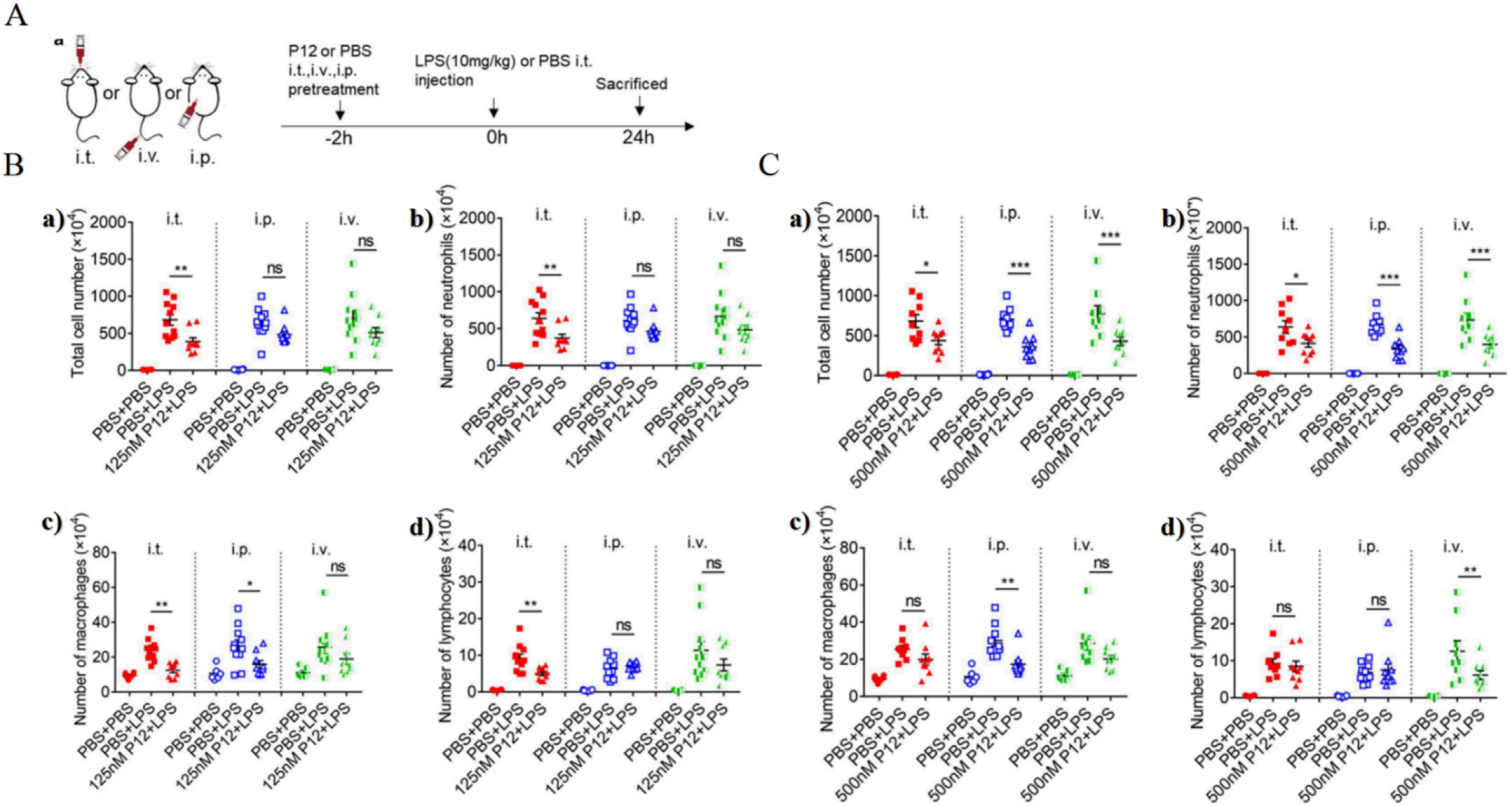
**A**. Schematic diagram of LPS-induced ALI model with the pretreatment of P12 (50 ​μL, 125 ​nM) or the same volume of PBS through i. t., i. v. or i. p. Administration 2 ​h before the LPS (10 ​mg/kg) or PBS injection (i.t.). 24 h after LPS injection, mice were sacrificed to harvest the BALF for the analysis of the number of total cells. **B**. The effect of administration routes on cell infiltration in the BALF in the LPS-induced ALI mouse with a lower dose of P12. Different cells for neutrophils (c), macrophages (d) and lymphocytes (e). N ​≥ ​6 per group, ns: not significant, ∗p ​< ​0.05, ∗∗p ​< ​0.01. **C**. The effect of administration routes on cell infiltration in the BALF in the LPS-induced ALI mouse with a higher dose of P12. Reproduced with permission from Ref. 83 Copyright 2021, Springer Nature.

Bao's team developed a unique nanosystem, P12, made of hexapeptides and gold nanoparticles (Fig. 3). P12 exerts a unique anti-inflammatory effect that attenuates multiple TLR signaling pathways and downstream inflammatory responses in macrophages. P12 can target lung macrophages, promoting their differentiation and ameliorating lung injury and inflammation in a mouse model of acute lung injury [126]. In this study, the therapeutic activity, biodistribution, and lung cell-targeting characteristics of P12 were investigated for intratracheal, intravenous, and intraperitoneal routes of administration and the following conclusions were drawn [127]. (1) The administration route of the drug can affect the therapeutic effect of P12 and is dependent on concentration. At higher concentrations, the three routes showed similar therapeutic effects. At lower concentrations, the local intratracheal injection of P12 into the lung had the best therapeutic effect. (2) Different administration routes have different biodistribution profiles. When P12 was administered by intrathecally, the drug accumulated most in the lungs and less in the liver and spleen. When P12 was administered intravenously, higher amounts of the drug accumulated in the liver and spleen. However, when P12 was administered intraperitoneally, the drug was more likely to accumulate in lymph nodes. Further, P12 biodistribution in different models was explored by establishing mouse models in the healthy and lipopolysaccharide (LPS) groups. The presence of acute pneumonia was found to affect P12 biodistribution. (3) After intrathecal administration, P12 was demonstrated to abundantly accumulate in the lung and specifically target lung macrophages. The other two administration routes did not exhibit this characteristic.

Most peptides are currently administered by injection. Injection offers the advantages of high bioavailability and definite curative effect. However, because of the complicated operation and low patient compliance associated with injections, new administration routes must be developed. Oral administration can improve patient compliance and has superior convenience, and therefore, the development of oral peptides is of great significance. However, considering the complex GI environment, this development needs to overcome multiple challenges [128]. In addition, more administration routes are being continuously researched and used for the administration of peptide drugs to improve the efficacy of drugs and achieve diversified and personalized treatment [129].

Because of the large nasal surface area, highly vascularized subepithelial layer, and rapid drug clearance, intranasal administration can be used for systemic drug absorption and is a feasible dosing regimen [130]. Nasally administered drugs are usually absorbed into the blood circulation and other tissues through the mucosa to exert local or systemic therapeutic effects. Compared with other administration routes, nasal administration can avoid the liver first-pass effect and the blood–brain barrier, presenting the advantages of fast absorption and high bioavailability [131]. The nasal cavity has an enzymatic degradation environment. To improve the stability of immunogens, nano-delivery systems are usually used for nasal administration. The nasal mucosa-related lymphoid tissue is rich in B cells, CD4^+^ and CD8^+^ lymphocytes, and dendritic lymphocytes. Therefore, nasal administration can effectively stimulate systemic and mucosal immune responses compared with intramuscular and subcutaneous injections [132]. In-depth research on the nasal mucosa has revealed that M cells distributed in the nasal mucosa are key players in the translocation of antigens and drugs. Therefore, establishing an *in vitro* nasal model based on M cell differentiation is beneficial for more accurately simulating the presence of drugs in the transport in the nasal mucosa [133]. Gao's team used Calu-3 ​cells to construct a nasal M cell model on an inverted co-culture model, which better reflects the real state of nasal M cells [134]. Furthermore, the team conducted related experiments through co-administration of OVA@PLGA and iRGD to evaluate the effect of the CPP iRGD via nasal administration [135]. The experiment found that nanoparticles had a certain distribution in the heart, liver, spleen, lung, and kidney through nasal administration. The distribution of nanoparticles in the deep nasal cavity was higher in the iRGD group than in the control group. Experiments showed that some nanoparticles enter the blood circulation and are distributed throughout the body. iRGD application increases the transport of nanoparticles and promotes their uptake by the nasal mucosa. In addition, nasal administration can better stimulate mucous immunity and enhance systemic immunity.

To prevent the aging of vaccines, they must be transported and stored in a cold chain at approximately 4 ​°C. The harsh transportation and storage conditions are associated with high transportation costs and the need for numerous professionals [136]. Sublingual vaccine delivery (under the tongue) requires no injection and has the potential for self-administration, with a higher potential for thermostability than conventional vaccines [137]. However, the development of sublingual biomaterial vaccines has been limited because of the delivery issues in the salivary mucus layer. Collier's team developed a sublingual nanofiber vaccine based on self-assembling Q11 peptides coupled to mucus-inert materials [129]. The prepared nanofibers were further used to produce a sublingually dissolvable SIMPL tablet vaccine [138]. A mixture of self-assembled peptide-polymer nanofibers, sugars, and adjuvants was lyophilized to produce the SIMPL tablet vaccine. The effect of SIMPL tablet-induced sublingual immunity on model epitopes of ovalbumin and clinically relevant epitopes of *Mycobacterium tuberculosis* was further investigated. In the experiment, by adding sugar excipients and using freeze-drying technology, SIMPL tablets with high porosity and good properties were produced. The tablets exhibited good sublingual solubility as well as thermal stability. A series of animal experiments revealed that the SIMPL tablet vaccine can increase the antibody response of the model epitope pOVA and the *M. tuberculosis* epitope ESAT6.

Recently, the immune function of the skin has gradually received considerable attention. Transdermal immunotherapy has been extensively focused on as a drug delivery method with high compliance and good durability. However, Nontheless, the hydrophobic structure of the stratum corneum can prevent the invasion of foreign molecules, which greatly limits the application of peptide- and protein-containing vaccines [139]. Hay fever, also known as allergic rhinitis and hay fever, is a symptomatic immunoglobulin E (IgE)-mediated allergy caused by exposure to pollen [140]. Allergen immunotherapy (AIT) is an effective treatment for hay fever. Subcutaneous injection (subcutaneous immunotherapy) and sublingual application using tablets or drops (sublingual immunotherapy) are the main administration routes used for the clinical treatment of AIT [141]. A simple and safe transdermal immunotherapy is proposed by Kong et al. [142]. The vaccine T cell epitope peptide containing pollen allergens was prepared as a solid-in-oil (S/O) nanodispersion system. These S/O nanodispersions are oil-based dispersions of nanoparticles and are formed by a coating of hydrophobic surfactant molecules, which can effectively overcome the hydrophobic structure of the stratum corneum in transdermal drug delivery [143]. The *in vitro* penetration test demonstrated that the T cell epitope peptide could effectively penetrate the skin. In vivo experiments using a mouse model of pollination disease, the S/O nanodispersions loaded with T cell epitopes were found to inhibit the production of serum antibody IgE and cytokines. The therapeutic effect in relieving allergy symptoms is comparable to that of subcutaneous injections.

## 4 Pharmaceutics applications of self-assembled peptides

### 4.1 Drug delivery

Conventional drug delivery usually suffers from solubility problems, burst release, and low bioavailability due to the high clearance or metabolism, high degradation and non-specific distribution [14]. Drug delivery systems can target drugs to specific tissues, thus reducing toxic side effects. Self-assembling peptides can form different nanostructures in response to the changes in the assembly environment, and therefore, differences in the pathophysiological microenvironment (including pH, enzymes, membrane receptors) are used to trigger the self-assembly and drug release to control cell fate, kill cancer cells, improve drug targeting to reduce the damage to normal cells, and develop appropriate immunogenicity and metabolic stability [144]. Peptide-based nanostructures have excellent stability and diversity, thus being promising in the field of drug delivery due to their chemical diversity, biocompatibility, and specific binding to target proteins (Table 3) [145].

**Table 3:** Application of short peptides in drug delivery.

Short peptide		Delivery form	Delivery Theory	Advantages	Ref.
peptide-drug conjugates (prodrugs, PDCs)	DOX-KGFRWR	Nanofibers	Covalently linked by peptide-drug, in response to the specific microenvironment (pH, enzymes, metabolites) of the diseased tissue	Precise spatial and temporal control during drug release achieved by harnessing external stimuli	[146]
	PDC-DOX_2_	Spherical micelles			[147]
	FA-GFK(Taxol)E-*s*-s-EE	Hydrogel			[148]
	NF-SS-MP	Nanofibers			[149]
short peptide-based soft materials	Azo-glutamine-phenylalanine-alanine hydrogel	Chiral hydrogels	Optical response for chiral transformation		[150]
	BPmoc-Ins	Supramolecular hydrogel	Addition of stimulus-triggered degradation units responds to specific stimuli and regulates the self-assembly process		[151]
Short peptide-based targeted delivery vehicles	Nap-FFYp	Hydrogel/Nanoweb	Cell-specific microenvironment triggers selective self-assembly of hydrogels around target cells	Targeting of tissues/cells based on specific stimulus responses that occur in response to changes in the spatial structure of the peptide at the preferred site	[152]
	L-1, D-1	Nanofiber			[153]
Sustained-release short peptide hydrogel	GV8	Hydrogel	Controlled drug release from degradable supramolecular hydrogels guided by concentration-dependent energy storage modulus	Sustained-release of drugs encapsulated in a hydrogel network with a microporous structure	[154]
	Fmoc-FFRRVR	Hydrogel	Hydrogel is easy to decompose in acidic environment with slow release ability		[155]

#### 4.1.1 Stimulus-responsive release

The main among the peptide-based therapeutics is the one that uses peptide-drug conjugates (prodrugs, PDCs), in which the drug is covalently modified with a specific peptide sequence to achieve the desirable pharmacokinetic characteristics. PDCs are formed by covalently linking a drug molecule to a peptide fragment with the advantages of a precise structure, high drug loading capacity and low drug leakage [2]. The combination of low molecular weight drugs and self-assembled short peptides forms supramolecular hydrogels, providing the precise spatial-temporal control through external stimulations. Among them, the enzyme-instructed self-assembly strategy offers an effective way to form nanofiber networks and results in hydrogels under overexpressed enzyme in malignant tumors. A hydrogel precursor (phosphorylated naphthalene-FFKY-succinic acid-taxol) was synthesized following this method. The process that leads to the transformation of the precursor into self-assembled nanofibers and provides a supramolecular hydrogel starts with the action of phosphatase in cancer cells. This hydrogel slowly releases the taxol into an aqueous medium. It indeed was the first time that the effectiveness of the enzyme-instructed self-assembly and hydrogelation of the prodrug was shown [156]. Similarly, Liu and coworkers designed a phosphatase-triggered self-assembled platinum prodrug from the same short peptide sequence (phosphorylated Nap-FFKY), which possesses significant antitumor growth effects on breast cancer and lower toxicity towards other major organs than the free cisplatin drug [157]. Wang et al. reported a DOX-KGFRWR able of self-assembling into long nanofibers that inhibit tumor growth in situ and prevent lung metastasis [158]. Recently, this team synthesized a novel PDC, such as PDC-DOX_2_, consisting of two adriamycin (DOX) molecules covalently linked to a short peptide with self-assembly function (KIGLFRWR), able of forming spherical micelles by hydrophobic interactions in neutral aqueous solution. The results revealed that PDC-DOX_2_ is the most stable spherical structure under neutral conditions and is feasible for intravenous injection [159]. Its morphology remains stable in a neutral environment, but aggregates as the pH decreases due to electrostatic interactions. It slowly releases the drug under phosphate-buffered saline (PBS) at pH 6.5 without releasing free DOX under normal physiological conditions. The micelles are coated with negatively charged natural polysaccharide shell hyaluronic acid (HA) to form a core-shell structure of the nanomedicine HA@PDC- DOX2 since the surface of the micelle is positively charged. HA can be used to modulate the particle size and stability of HA@PDC-DOX2 to enhance the targeting of PDC-DOX2 micelles due to its interaction with the overexpressed receptors in cancer cells.

The redox difference between normal cells and tumor cells has been extensively investigated for designing stimulus-sensitive supramolecular nanofiber hydrogels [160]. Tumor cells overproduce intracellular glutathione (GSH) than normal cells, resulting in a strongly reducing environment. The disulfide bond can be used in response to the GSH stimulus and serves as a cleavable linker to connect self-assemble short peptides and the therapeutic parts. Yang et al. designed a series of molecular hydrogels of FA-Taxol conjugates based on the disulfide bond. FA-GFK(Taxol)E-*s*-s-EE was first synthesized as a precursor of the gelator and subsequently peptides of GKE, KE, and K were used instead of GFKE. All designed FA-Taxol conjugates form hydrogels, which sustain the release of Taxol through ester bond hydrolysis and inhibit tumor growth more efficiently, compared with Taxol [161]. Liang et al. synthesized a prodrug of NF-SS-MP by linking 6-MP to a short naphthylacetic acid terminal peptide (Nap-FFYE, NF) with disulfide bonds, which self-assembles to form nanofibers in neutral aqueous solution under the influence of hydrogen bonding and electrostatic interactions [162]. The nanofibers exhibit a sensitive glutathione response and controlled release properties due to the high sensitivity of the disulfide bonds to reducing conditions. The encapsulated 6-MP shows remarkable killing effects in initial incubation time and is able to significantly reduce the toxicity to normal cells after 24 ​h incubation, showing the advantages of low toxicity and high biocompatibility.

The use of other anticancer drugs expands the feasibility of short peptide-based hydrogelators of anti-cancer drugs and develops more of these hydrogels for combined therapy. Huang and coworkers designed hydrogels formed by azobenzene (Azo)-peptides, possessing a photo-response property, which was realized based on the E−/Z-transition of the conformation upon light irradiation. The Azo-glutamine-phenylalanine-alanine hydrogel is a promising photo-responsive soft material for the controlled release of drugs [163]. Other stimulus-responsive supramolecular hydrogel systems focus their attention on soft materials due to their potential applications in the pharmaceutical area. In these systems, the addition of the stimulus-triggered degradation units in the N-terminal peptides can respond to a specific stimulus to form the self-assembled nanofiber network [164]. The conjugation of dipeptide and *p*-borono-phenylmethoxycarbonyl (BPmoc) degrades p-quinone methide and CO_2_ in the presence of H_2_O_2_, breaking the vital intermolecular interactions between the peptide residues, finally resulting in the destruction of the supramolecular hydrogel [165]. According to this mechanism, the synthesized BPmoc-FF hydrogels encapsulate glucose oxidase and trigger the insulin release under the oxidation condition. Another two different types of reduction- or photo-responsive supramolecular hydrogels are also prepared by covalently attaching the FF to the p-nitro-phenylmethoxycarbonyl (NPmoc), or to the 6-bromo-7- hydroxycoumarin-4-ylmethoxycarbonyl (Bhcmoc) group.

#### 4.1.2 Targeted and sustained release

The fact that the anticancer therapeutics work through the self-aggregation of non-toxic short peptides inside or around cancer cells has recently become a hot topic. Xu group developed a range of self-assembled short peptides for anti-cancer applications. Among these, one typical study reported a phosphorylate precursor (Nap-FFYp) of a hydrogelator. The phosphatases oversecreted by cancer cells dephosphorylate a precursor to trigger the self-assembly of the hydrogelator to result in pericellular hydrogel/nanonets selectively around the cancer cells. The cancer cells are killed since the pericellular hydrogel/nanonets block the cellular mass exchange [44]. Except for high-expressed extracellular phosphatases, Xu designed and synthesized small peptide precursors as intracellular substrates of carboxylesterase. The precursors from the peptides transformed into self-assemble nanofibers through the intracellular carboxylesterase. At the optimal concentrations, the precursors significantly increase the activity of cisplatin against the drug-resistant ovarian cancer cells, but themselves not harm the cells [153]. D-peptides are gaining increased attention due to their stability. Since the biological use of d-amino acids is rare, the cellular uptake of D-peptides is ineffective. Xu et al. developed taurine, a natural amino acid, covalently connected with D-peptides. The ester conjugates of taurine and D-peptide are able to enter cells through both dynamin-dependent endocytosis and macropinocytosis, contributing to intracellular molecular self-assembly by the induction of intracellular esterase [166]. Other than short peptides, other non-toxic therapeutics including aromatic carbohydrate amphiphile [167], cholesterol conjugates [168], and biomacromolecules [169] can also generate cytotoxicity through the self-aggregation inside or around cells.

**Fig. 4:**
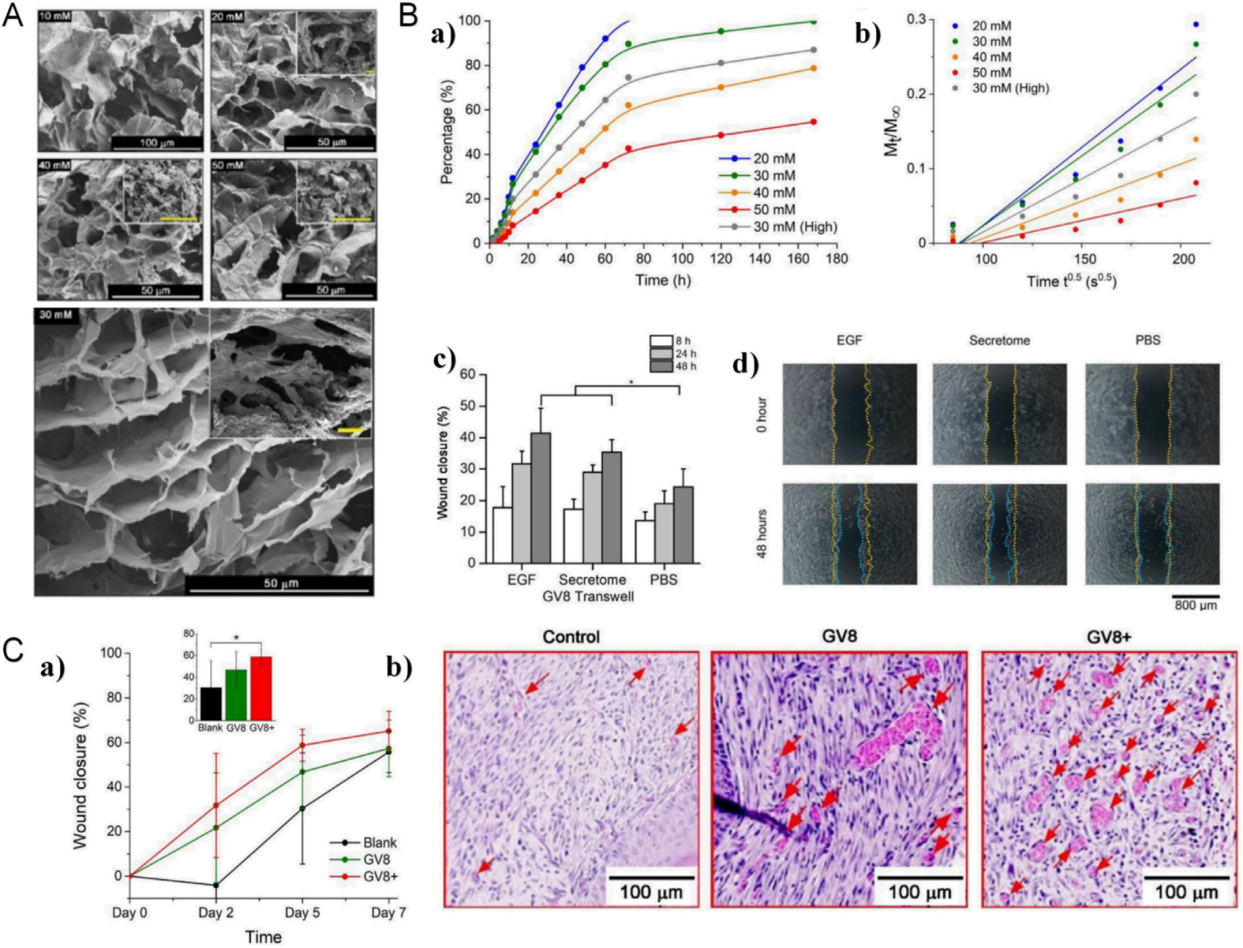
A. FESEM images of freeze-fractured cross-section of GV8 hydrogels prepared at peptide concentrations ranging from 10 to 50 ​mM. Yellow scale bars in inset images ​= ​100 ​μm. **B.** Release profiles(a) and Mt/M∞ vs t0.5 diffusion plot of encapsulated secretome (b) at different encapsulation concentration. Wound closure (%) of scratch wound assay(c) measured over 48 ​h with encapsulated EGF and secretome released with GV8 hydrogel transwell setup. Representative images of HaCaT cell migration(d) at 0 ​h and 48 ​h (initial scratch boundaries-yellow dotted lines; cell boundaries after 48 h-blue dotted lines). **C.** (a) Average wound closure area measured for n ​= ​3–5 samples at day 5. (b) Regions from each sample group were selected to show areas with the highest microvessels densities in H&E-stained skin sections of the wounded area at day 7. (Microvessels are indicated by red arrows) Reproduced with permission from Ref. 118 Copyright 2020, Elsevier. (For interpretation of the references to colour in this figure legend, the reader is referred to the Web version of this article.)

Supramolecular hydrogels present similar properties to extracellular matrices in tissues, being soft, biologically active, with mostly water-filled micropores, making them excellent carriers for drug delivery [170]. Therefore, drug encapsulation in a hydrogel network can potentially realize a slow release, or drug binding to peptides can be used for a slow release of the drug through the protein hydrolysis of the hydrogel. Wang et al. performed a study to find that the bionic octapeptide GV8 can self-assemble under mild conditions to form degradable supramolecular hydrogels with high energy storage modulus and good biocompatibility, which can sequester single or multiple macromolecular protein-based therapeutic drugs and showing concentration-dependent energy storage modulus and controlled drug release. Hydrogels loaded with adipose mesenchymal stem cell secretome maintain the mechanical strength while maintaining a continuous supply of the above secretome by maintaining the secretory activity, demonstrating the potential of GV8 peptide hydrogels as carriers for the encapsulation and delivery of macromolecular drugs (Fig. 4) [171]. Lian et al. encapsulated HRP in Fmoc-FF hydrogels to enable the in situ monitoring of hydrogen peroxide released from HeLa cells. Fmoc-FF serves as both a matrix embedded enzyme model and a substrate for cell adhesion. HRP is immobilized within the hydrogels in a stable manner and keeps intrinsic bioactivity towards H_2_O_2_. As a result, the peptide hydrogel has good reproducibility, high stability, and selectivity [172].

Li et al. revealed that Fmoc-FFRRVR can be prepared as a hydrogel after simple mixing with aqueous solution in powder form compared with Fmoc-FF, which possesses good cell uptake, enhanced cell permeability and retention, and good biocompatibility due to its amphiphilic nature [173]. The hydrogel loaded with DOX performs a sustained release of the anticancer drug DOX, with the hydrogel maintaining its properties when high concentrations of DOX are added. Moreover, the *in vitro* release experiments at different pHs showed that the Fmoc-FFRRVR hydrogel is easily decomposed in acidic solutions and has a long release ability. In conclusion, Fmoc-FFRRVR can be used as a carrier for a better entrance of DOX into cells and targeted delivery of hydrophobic DOX to the nucleus of cancer cells, achieving the slow and controlled release of the drug [155].

Peptide self-assembly still faces problems as a scaffolding material for drug release, such as susceptibility to physiological environment and less than ideal structural stability; thus, breakthroughs and solutions are urgently needed [145]. Although many forms of peptide assembly exist, it is still challenging to predict and regulate the molecular structure of peptides directly and accurately. The continuous efforts of researchers and the availability of smarter and more accurate instruments allow the continuous visualization and discovery of peptide structures. Although the exploration and application of peptide-based nanomaterials is still a challenging task, their future research prospects are broad and bright.

### 4.2 Antibacterial activity

Antibiotic resistance has become an increasingly serious problem due to the misuse of antibiotics and the continuous evolution of microorganisms. The emergence of nano-antimicrobial agents with properties such as controlled drug release, circulation and targeted drug delivery has brought hope to solving the problem of the multidrug resistance of microorganisms. Most of the known antimicrobial peptides have the following characteristics: 1. They are mostly cationic peptides [174]. 2. The secondary structure affects the antimicrobial activity (The secondary structure is the one involved in the antimicrobial activity) of the self-assembled peptides [175]. 3. The nanostructures formed by the self-assembly of short peptides have excellent and unique antimicrobial properties [176].

Glossop et al. synthesized a battalion with a special structure, such as HG2.67, which is a pentapeptide obtained by replacing the hydrophobic Leu-D-Phe dipeptide of battalion with dinaphthylalanine (Nal), while the acyl group was replaced by Fmoc while retaining the N-terminal D-2,4-diaminobutyric acid (D-Dab) residue. The structural modifications to obtain HG2.67 with superior amphiphilic properties and enhanced intermolecular π-π stacking lead to the ability of HG2.67 to be rapidly assembled into self-supporting hydrogels in phosphate-buffered saline at 1% w/v concentration. In terms of applications, the hydrogels exert enhanced antibacterial activity, offering the possibility of further development for topical antibiotic therapy, slow-release antibiotics, and drug delivery [177]. Some studies found that the combination of antibiotics with short peptides to form prodrugs improves the stability, efficacy, and safety of antibiotics. McCloskey et al. found that naproxen-modified FFKK supramolecular hydrogel (NpxFFKK) possesses excellent antibacterial and anti-inflammatory properties in a tetrapeptide sequence of diphenylalanine di-lysine (FFKK–OH) capped with three NSAIDs at the N-terminal end with racemic ibuprofen, indomethacin, or (S)-(+)-naproxen [178]. The combination of chloramphenicol succinate, a soluble prodrug of chloramphenicol, with diglycine (GG) to form CLsuGG to be used against gram-negative bacteria was also reported by Wang et al. [179]. CLsuGG inhibited the activity of *E. coli* tenfold more than CLsu by accelerating the hydrolysis of bacterial lactone bonds; CLsuGG also exerts a lower cytotoxicity to bone marrow stromal cells. This study is a clear example of improving the bacterial activation rate of antibiotic precursors, providing an idea for future studies to increase the antibacterial capacity by increasing the intracellular accumulation of antibiotics, consequently helping the improvement of the safety and efficacy of the existing antibiotics [180]. Some Trp-rich antimicrobial peptides have the tendency to interact with bacterial paper membranes. Zhang et al. designed and synthesized three peptides, FWYp, WWYp and WFYp using tryptophan and investigated their antimicrobial properties using *Escherichia coli* (ATCC 25922) and *Staphylococcus aureus* (ATCC 12600) as model cells [181]. The bacterial suspensions of FWYp, WWYp and WFYp induce significantly higher flocculation rates due to the generation of self-assembled nanostructures and enzyme-triggered dephosphorylation leading to increased cationic charge density, interacting with lipid membranes under electrostatic and hydrophobic interactions. Moreover, they induce the external mode penetration of bacteria and depolarization of the cytoplasmic membranes, thus exerting significant antibacterial properties and concentration-dependent bactericidal activity. Ma et al. studied the self-assembled structures of two constitutively heterogeneous peptides, Ac-RFR-NH2 and Ac-SFR-NH2 and investigated the antibacterial activity of both [182]. The antibacterial effect of the self-assembled peptides is related to the surface potential of the peptide nanostructures and the self-assembled morphology affecting the binding affinity to bacterial cell membranes. In addition, linear short peptides with basic residues at the end of the sequences facilitate the formation of better self-assembling nano-antimicrobial peptides.

Diphenylalanine nano self-assemblies are potential antimicrobial agents and are less likely to develop resistance than cationic short peptides when applied to bacteria due to their characteristic hydrophobicity and non-cationic nature [174]. The smallest self-assembled dipeptide of antimicrobial supramolecular polymers FF was proposed by Schnaider et al. [183] as neutral and aromatic compared with classical antimicrobial peptides, which are usually amphiphilic and cationic. The FF nano-self-assemblies inhibit bacterial growth, trigger a stress response leading to the upregulation of regulatory proteins, induce the disruption of the bacterial morphology, and induce membrane permeation and depolarization, showing a significant antibacterial activity. FF exhibits a significant biocompatibility and specific toxicity to prokaryotic cells, and the bacterial membrane is the main target of the FF nanostructure.

**Fig. 5:**
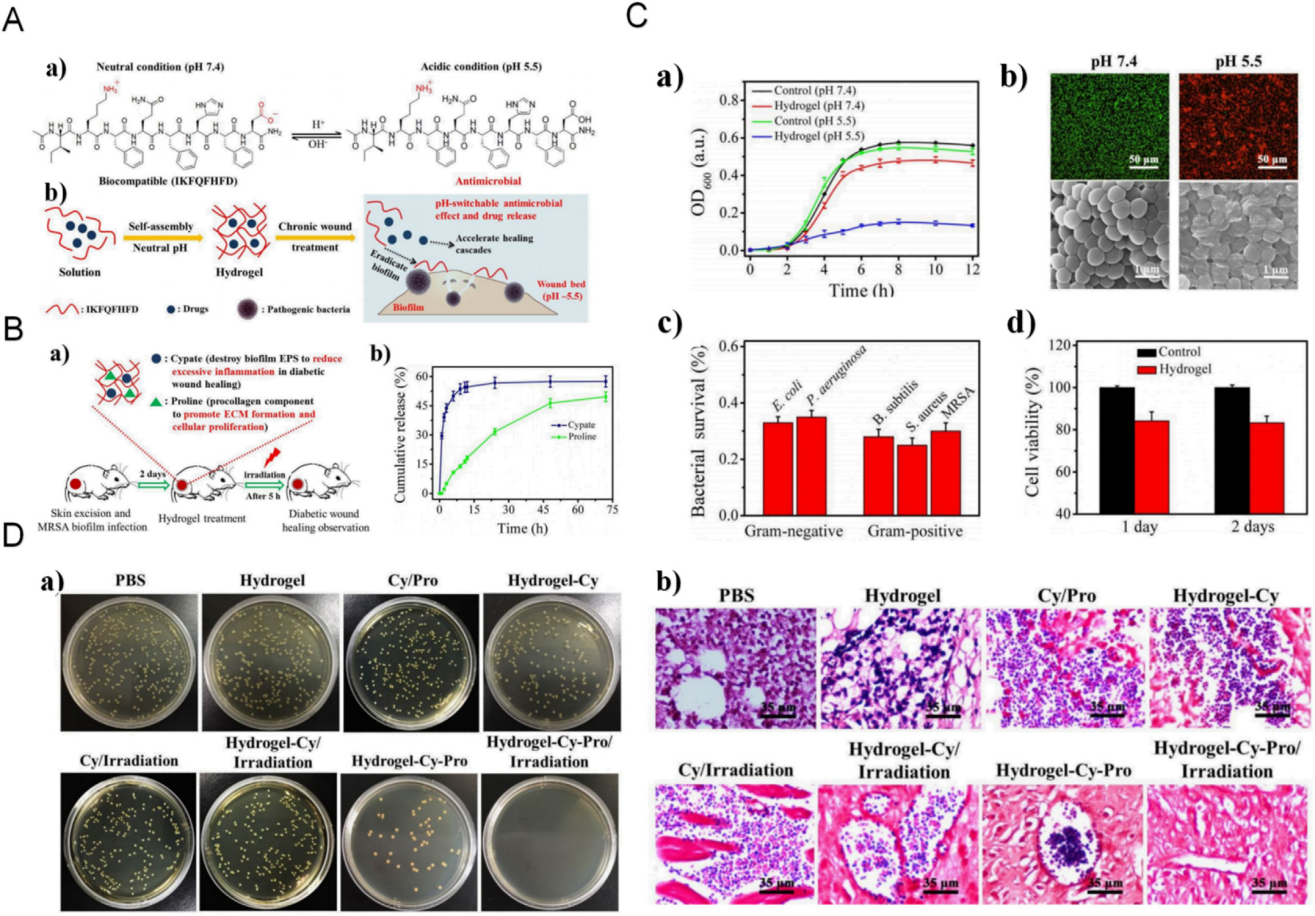
A. (a) Changes in the chemical structure of IKFQFHFD under neutral and acidic conditions. (b) pH-switchable antimicrobial IKFQFHFD-based nanofiber networks for biofilm eradication and rescuing stalled healing in chronic wound. **B.** (a) Integrated hydrogel-Cy-Pro system for biofilm eradication and rescuing stalled wound healing in diabetic mice. (b) The dosage of IKFQFHFD, cypate and proline in the integrated hydrogel-Cy-Pro system was 10 ​mg/mL, 5 ​μg/mL and 20 ​mg/mL. Release curves of cypate and proline loaded in the hydrogel-Cy-Pro system (pH 5.5). **C.** (a) Growth curves of *S. aureus* incubated with hydrogel under different pH conditions. (b) Under different pH conditions, representative SEM and overlapping fluorescence images for the live/dead staining of *S. aureus* incubated with hydrogel. (c) The antimicrobial effects of hydrogel toward gram-negative bacteria of *E. coli* and *P. aeruginosa*, and gram-positive bacteria of B. subtilis, *S. aureus* and MRSA (pH 5.5).(d) Viability of HUVEC cells after incubation with hydrogel for 1 and 2 days (pH5.5). D (a) Bacterial culture from the skin tissues of diabetic mice wounds infected with MRSA biofilm. (b) The corresponding gram-staining images of the skin tissues of MRSA biofilm-infected diabetic mice wounds. Reproduced with permission from Ref. 131 Copyright 2019, American Chemical Society.

Recently, synergistic strategies such as combined photothermal therapy and photodynamic therapy have become new hot research to establish multifunctional drug delivery protocols to reach better and more efficient sustained antimicrobial effects. Wang et al. designed an octapeptide that forms a dynamic supramolecular hydrogel mediated by pH and loaded with cytosine proline, and used a synergistic strategy of antimicrobial hydrogel, photothermal therapy, and proline to eradicate biofilms to promote chronic wound healing (Fig. 5) [184]. Since the cations of antimicrobial peptides, as well as hydrophobic residues, determine their non-specific action and disrupt bacterial membranes in a unique way, thus producing antibacterial effects, the emergence of self-assembling antimicrobial peptides provides new possibilities for the further development of antibacterial drugs. Zhang's group reported a photodynamic antimicrobial therapy based on the remarkable superiority of photodynamic therapy on the treatment of a wide range of drug-resistant bacteria, reducing the propensity for antibiotic resistance [185]. A hybrid hydrogel consisting of 20 nanofibers and nanoparticles was designed by combining the amphiphilic peptide Fmoc-FF with the fullerene derivative C60-PTC. The generation of ^1^O_2_ by this hybrid hydrogel results in a photodynamic antibacterial effect compared with the enhanced C60-CPT aggregates thanks to the interaction between C60-PTC and nanofibers, and the hydrogel slowly degrades to 87% in 8 days under simulated blood flow conditions, demonstrating the sustained antibacterial effect of this composite hydrogel in vivo. This discovery establishes a multifunctional nanoplatform for targeted sustained drug delivery of fullerenes for photodynamic antimicrobial therapy, providing new ideas for targeted, slow and controlled release of drug by delivery systems potentially used as antimicrobial therapy.

### 4.3 Stabilizers

The self-assembled short peptides have been investigated as carriers for the stabilization and delivery of drug molecules. A single dipeptide (FF) had been presented as a hydrogelator. A study demonstrates that the emulsions remain stable for months due to the addition of dipeptide amphiphiles. The surfactants have been extensively utilized in traditional emulsion systems to avoid phase separation and demulsification under physiological conditions. However, surfactants have the disadvantages of biotoxicity and limited stability. The self-assemble dipeptides, as a novel interfacial stabilizer, rapidly form nanofibrous networks at the organic/aqueous interface through π-π stacking and hydrogen bonding. This network promotes a stable encapsulation under specific temperature, salts and enzyme conditions [186]. 2NapFF has been most extensively studied, and one method of synthesis is the preparation of self-assembly of dipeptides at the air-water interface by drop-casting with the support of protonation of the carboxylic acid group of the dipeptide to form a local elastic sheet. Hydrogels can also be formed for stabilizing aqueous foams by adding metal ions to 2NapFF [187]. Aviño et al. investigated the properties of self-assembled hydrogels formed by the dipeptide 2NapFF at different concentrations of salt (MgSO_4_) and developed a repetitive gel formation process accordingly. The hydrogels are disrupted and deformed under high shear covering bubbles or droplets, and the maturation of bubbles is prevented, thus acting as stabilizers. The hydrogels also possess low stabilization capacity at low concentrations and high stability to emulsions prepared from oil [188].

Ricardo et al. discuss the emulsification behavior of self-assembled peptides, the influence of the molecular structure and the conditions of the system on the function of peptide-stabilized emulsions, the design and discovery of bands with stabilizing properties and they finally present the outlook and challenges of the field. The article mentions the unique characteristics of peptides that can be used as emulsifiers compared with commercial surfactants such as sodium dodecyl sulfate (SDS): 1. A small molecular size makes them easy to design, predict, and tune. 2. The important property of stabilizers as emulsions is amphiphilicity, and the complexity of factors affecting amphiphilicity of peptides and their vulnerability to the environmental conditions complicate their analysis. 3. The evaluation of the hydrophilic and hydrophobic properties of the peptide is more complex, due to the more complicated relationship between the secondary structure of peptides and amino acid sequences [189]. Scott et al. reported that a combined experimental/computational approach predicts tripeptides able to stabilize emulsions. The coarse grain molecular dynamic simulation is a useful tool in evaluating the molecular self-assembly of supramolecular materials [190]. By leveraging this strategy, the specific sequence of tripeptides shows a certain emulsion-stabilizing ability at both ambient and high temperatures. Ulijn et al. selected some tripeptides with good aggregation from a preliminary screening of 8000 tripeptides and finally KYW, KFF, KYF, FFD and DFF were selected for the study [191]. Their results demonstrated that the tripeptide-based emulsions are relatively stable with respect to temperature, pH and emulsion concentration. The emulsions are divided into two different layers by the increasing temperature, and the de-emulsified positive state is observed at 60 ​°C for all samples except KYF, which remains in the emulsified state at this temperature. This indicates that KYF has higher heat resistance than the other samples and provides an idea to study the regulation of heat resistance by the cationic modification of peptide sequences. Bai et al. investigated the self-assembly of a series of dipeptide or tripeptide compounds with different hydrophobicity and functional groups, such as tyrosine-leucine (YL), tyrosine-alanine (YA), tyrosine-serine (YS), FF and triphenylalanine (FFF) modified by Fmoc or pyrene at the organic/water interface to emulsify and stabilize water-in-oil or water-in-oil emulsions. The results showed that highly stable microencapsulated fiber networks are rapidly formed in the organic/water environment by aromatic π-π stacking and hydrogen bonding in the sequence and used to stabilize organic droplets in aqueous media (or aqueous droplets in organic media) where the nanostructured network of Fmoc-YL is mainly in the aqueous phase, while Fmoc-YA forms gels in buffered solutions and chloroform (30 ​mM). Fmoc-YS only forms gels in aqueous media. Further experiments showed that Fmoc has excellent emulsion thermal stability, is not affected by salts and can be digested by suitable enzymes to stabilize the membrane [186].

Based on previous reports of examples of aromatic amino acids self-assembling as emulsifiers in short peptides [192], Scott et al. explored the possibility of using tetrapeptides entirely formed from genetically encoded amino acids to stabilize emulsions [193]. A series of experiments were conducted using five tetrapeptides, PTAL, HGII, LQCS, LSQV and RRET, and among them HGII (polar head - hydrophilic histidine, hydrophobic tail -glycine, isoleucine) shows the most significant adsorption to the water-air interface and the lowest surface tension at 30 ​mm, exhibiting a low molecular weight surfactant behavior due to the amphiphilic molecular structure, which results in a superior surface activity. Apart from the above results, scientists also reported that the self-assembled short peptides are able to be bio-catalyzed to stabilize emulsions. The phosphorylated precursors prepared by Moreira et al. can be changed into self-assembling aromatic peptide amphiphiles (Fmoc-tyrosine-leucine, Fmoc-YL) by alkaline phosphatase, which form networks at the biphasic organic/aqueous interface. The storage of the solvent mixtures with phosphorylated peptides for one month resulted in the activation of the emulsion after phosphatase addition and shaking [194]. Furthermore, Castelletto et al. investigated the novel surfactant-like peptide (Ala)9-Arg (A9R) with multifunctional self-assembly and bioactive properties, which is able to form hydrogels in a pH-stable state and exerts antimicrobial activity and stabilizing properties to stabilize water/oil emulsions. The surfactant-like peptide (Ala)9-Arg (A9R) forms β-sheet fibers when the critical aggregation concentration exceeds during on-demand emulsification by protease action, and the water-in-oil emulsion is stabilized by the β-sheet fiber coating around the emulsion droplets [195].

### 4.4 Imaging agents

The development of molecular imaging is of great help for reasonably designing the peptide sequences and understanding the supramolecular self-assembly behavior during biological processes [196]. Among the various imaging modalities, fluorescence is a widely used imaging approach, because of its easy access, low damage, and high spatial resolution. The most common fluorescence application in peptide-based biomolecules is Fmoc due to the intense excitation at 270 ​nm and the corresponding emission at 320 ​nm. Zelzer et al. reported a fast, inexpensive and nondestructive fluorescence-based method, represented by the construction of short amino acid sequences monitored through a solid-state fluorescence. The attachment of Fmoc amino acids and the subsequent Fmoc deprotection are confirmed due to the fluorescent nature of the Fmoc group [197]. Moreover, the change in the position of the fluorescence peak of Fmoc is used to characterize the self-assembling mechanism of Fmoc-modified short peptide. In one study, Fmoc-modified 5-aminopentanoic acid (Fmoc-5), similar to Fmoc-FF, is used as a simple non-peptide model that confers great self-assembling properties [198]. The phenomenon of a dominant fluorescent emission peak at 324 ​nm at pH 2.0 and a step-like absorbance from 260 to 300 ​nm proved that Fmoc-5 molecules form 2-D quantum-well confined structures. The inherent optical properties of Fmoc at pH 10.0, an emission peak at 467 ​nm and a quasi-continuous absorbance, emerged because of Fmoc-5 hydrolyses. In addition to Fmoc, Zhang et al. also reported the supramolecular metallo-hydrogelator, combining a tripeptide derivative with ruthenium-(II) tris (bipyridine) complex ([Ru (bipy)_3_]^2+^). The long fluorescence lifetime of the [Ru (bipy)_3_]^2+^ derivative may elucidate the interactions of the molecular nanofibers with other molecules, facilitating the applications of supramolecular metal materials [199]. Kim and coworkers designed and synthesized a lysine dipeptide functionalized with 7-(diethylamino)-3-coumarin carboxylic acid (7-DAC), which is a fluorescence chromophore. The coumarin moieties in the self-assembled dipeptides are cross-linked at 365 ​nm irradiation, and the mechanical stability of hydrogel is enhanced [62].

The fluorescent peptide-based supramolecular hydrogel that is desirable for biomedical purposes evaluates into both the physiological and pathological processes in a real-time and sensitive manner. The perplexing question for the development of this system containing a fluorophore is that it is difficult to distinguish individual fluorescent hydrogelators and the corresponding nanofibers. Xu’ group did tremendous efforts to solve this dilemma. In one study, the author designed and synthesized a precursor NapFFK(NBD)Yp, a combination of the ﬂuorophore 4-nitro-2,1,3-benzoxadiazole (NBD), and a phosphate group on the tyrosine residue. They confirmed that self-assembled fluorescent hydrogelators are located in the endoplasmic reticulum inside live cells by imaging enzyme-triggered self-assembly of small molecules inside live cells. Moreover, they found the growth of the nanofibers from the endoplasmic reticulum towards the edge of the cells [200]. In another study of this group, four fluorescent peptide molecules were used to explore their distribution in a cellular environment [201]. This work illustrated that the different structures of the small molecules correspond to significant changes in the self-assembly. The distribution of the assemblies and their interaction with cellular components can be beneficial or detrimental to the cell. Min et al. proposed a covalent self-assembled peptide hollow nanocapsules and peptide sheets with tyrosine-rich short peptide YYYY to synthesize fluorescent nanocapsules and independent peptide films with certain mechanical strength by a one-step photopolymerization method [202]. The density of tyrosine radicals increases with pH and ionic strength, which in turn activates photopolymerization to form a nanosecret structure by tightly cross-linking the nanofilms. During further UV irradiation, the self-assembled morphology changed from incomplete spheres with pinholes to semi-capsules covered with highly cross-linked double tyrosine bonds over time to finally form hollow nanocapsules, which emit blue fluorescence at λ ​= ​410 under UV irradiation (λ ​= ​300 ​nm) in a pH 10 buffer to stimulate the cross-linking reaction of double tyrosine.

When a fluorescent structure is combined with a short peptide to form a nanostructure with fluorescent properties, it shows excellent performance in the field of cell imaging and cell tracking. Wang et al. reported that Rhodamine B, as a new fluorescent capping group, was bound with short peptides to form a precursor. The fluorescent nanofiber suspension via disulfide bond reduction confers a superb performance in the long-term cell tracking and tumor imaging applications [203]. Li et al. designed and synthesized the biocompatible amphipathic peptide C-3 (Arg-Val-Arg-Arg-Arg-Phe-Phe-Phe-NBD) consisting of a RVRRFFF sequence and a NBD fluorophore that can self-assemble into stable fluorescent nanomicelles to enable peptide nanosensors for cellular imaging. C-3 exhibits good thermal and acid-base stability (at a pH range of 3–9) [204]. C-3 has the ability to specifically monitor furin, a protein translocase overexpressed in tumors. The co-localization assay by Golgi Tracker Red revealed that C-3 binds to furin in the Golgi apparatus of MDA-MB-231 ​cells and achieves long-term tracking, which in turn can be used as a fluorescent probe for long-time detection of furin in live cell imaging, making it an effective tool for detecting specific cancer cells. The new supramolecular structure is the nanovesicle [198]. Dibromobiphenyl is a thiol-selective fluorescent imaging agent reused to cross-link peptides containing cysteine and homocysteine, with the resulting bisalkyl linker serving as a structural bond and fluorescent label. Horsfall et al. performed the macrocyclization of nine short peptides containing two cysteines and/or homocysteine on resins and in buffered aqueous solutions to obtain macrocyclic atoms of different sizes, and a bisalkyl linker was used as a thiol-specific fluorescent marker and structural constraint to proceed the study [205]. All these double alkyl cyclized peptides (peptides 1–9) show good photostability, and the double alkyl-constrained peptides 7, 9 and 10 have distinct fluorescence signals (385 ​nm, 385 ​nm, and 477 ​nm, respectively) that distinguish them from endogenous fluorescent molecules. Thus, they can be potentially used as effective tools to probe the secondary structure of short peptides and as fluorescent labels in cell experiments.

### 4.5 Combined peptides in bioengineering

#### 4.5.1 In situ self-assembly based on peptides

The physical and biochemical properties and functions of peptides are highly correlated with their supramolecular nanostructures. The construction of different peptide self-assembled nanostructures in situ not only endows peptides with structural functions such as improved stability and mechanical strength, but also possess superior activity [206].

Complicated physiological conditions in vivo affect the morphology and structure of self-assembled nanomaterials consequently affecting their properties at the biological interface. Therefore, a more in-depth and systematic study of the supramolecular behavior of self-assembled peptide-based nanomaterials under physiological conditions is needed to rationally link their uni-structures with self-assembled superstructures. By further observing and understanding the self-assembly and transformation processes under complex physiological conditions, we can further precisely regulate the non-covalent interactions in specific regions in vivo to overcome the uncontrollable stability of self-assembled nanomaterials and finally achieve in situ construction and transformation. The suitable physicochemical properties of bionic materials under complex physiological/pathological conditions are the key to their ability to mimic ecological processes in vivo becoming a hot research topic in many fields such as material science, chemistry, biology and medicine [207]. Natural macromolecules, represented by proteins, peptides, lipids and synthetic macromolecules, exhibit superior application potential with outstanding bioactivity, biocompatibility and process mimetic properties [208]. Self-assembly in vivo refers to constructing peptide nanostructures in the focal site in vivo by a “response switch” to optimize their biodistribution and reduce off-target toxicity. Human epidermal growth factor receptor 2 (HER2) is overexpressed in breast cancer. HER2 receptor dimerization leads to activation of downstream signals resulting in proliferation and survival of the malignant phenotype [209]. Zhang et al. designed a peptide (BP-FFVLK-YCDGFYACYMDV) capable of self-assembling into micelles in water. When the micelles specifically target HER2 conjugated on cancer cells, they are converted into nanofibers that disrupt HER2 dimers and block the expression of proliferation and survival genes in the nucleus, leading to apoptosis of cancer cells [210].

The extracellular matrix (ECM) is a dynamic hydrogel of interwoven protein fibronectin, comprising fibronectin (FNs) and laminin (LN) [211]. Protein FNs bind to cells through ligand-receptor interactions modifying the mechanical changes between the cytoskeleton and the ECM，playing an important role in regulating morphology, differentiation and proliferation, and adhesion of ECM [212]. The primary function of LN is to provide critical functions in the assembly of the basement membrane, acting as the main structural component to form network structures on the cell surface and anchor the cells to the basement membrane. Tumor invasion and metastasis are associated with enzymes (matrix metalloproteinases (MMPs)) overexpressed at the tumor site that degrade the extracellular matrix (ECM) [213]. Hu et al. constructed an artificial extracellular matrix (ECM) based on translatable laminin (LN)-mimetic peptide 1 (BP-KLVFFK-GGDGR-YIGSR) for the inhibition of tumor invasion and metastasis. The peptide is designed based on the unfolding principles of natural ECM formation to achieve in situ self-assembly. Peptide 1 consists of a hydrophobic bispyrene (BP) unit for forming and tracing nanoparticles, a KLVFF peptide motif for forming and stabilizing fibril structures through intermolecular hydrogen bonding, and a Y-type RGD-YIGSR motif as a ligand. Peptide 1 forms nanoparticles (1-NPs) via hydrophobic forces. When 1-NPs accumulate at tumor sites, they are transformed into nanofibers (1-NFs) which are able to bind to receptors on the surface of tumor cells and form AECMs to achieve effective inhibition of tumor metastasis. This in vivo self-assembly strategy provides a meaningful exploration for the design of effective drug-free biomaterials to inhibit tumor invasion and metastasis [214].

In situ self-assembly allows intelligent monitoring of biological processes, biomolecular activity and in vivo disease diagnosis. Nanostructures constructed in situ may interact with biomolecules within cells, cell surfaces and extracellular matrices, affecting the structure and function of cells and/or the microenvironment of specific regions. Compared to traditional chemotherapeutic drugs, physical therapy and other medications, in situ constructed nanomaterials possess potential therapeutic capabilities. Peptide biomimetic materials have been used in the fields of anti-tumor, antibacterial, and tumor imaging [215,216].

#### 4.5.2 Injectable hydrogel formed by peptides

Injectable hydrogels have advanced biomedical applications in the fields of tissue engineering, void fillers in surgery, bioadhesives or anti-adhesives [217]. In vivo biodegradability is a key issue, since drug delivery and cell adhesion or regeneration are influenced by the rate of hydrogel degradation. Therefore, hydrogels must be entirely biocompatible and biodegradable. In addition, a controllable nature, good targeting and retention properties and superior stability are essential considerations when designing injectable hydrogel materials. Self-assembled peptides capable of forming physical hydrogels are commonly amphiphilic dipeptides, especially those containing Fmoc or Naphthalene (Nap) [218]. With their ability to self-heal in certain conditions, they serve many important biotechnological applications [219,220].

However, the injectability of these gels are less studied. Some studies have shown that Fmoc-FF undergoes phase separation during the injection process [221]. Researchers have developed composites or hybrid hydrogels containing different organic or organic and inorganic materials to overcome the above problem [217].

Studies have shown that magnetic hydrogels exhibit a greater degree of controllability in achieving remote control of the mechanical or physical properties of the hydrogel, which is more conducive to the compatibility of the material with in vivo applications [222]. Comprising magnetic nanoparticles (MNP) in a hydrogel matrix to produce magnetic hydrogels broadens the scope of these materials in biomedical research. Embedded MNPs offer the possibility to achieve remote, on-demand modulation of the physical properties of hydrogels by applying an external magnetic field. The embedded MNPs irreversibly alter the mechanical properties of the hydrogels, as well as the microporosity and macroporosity of their three-dimensional (3D) structure, providing the potential to induce anisotropy. Mañas-Torres et al. investigated the behavior of Fmoc-diphenylalanine (Fmoc-FF) and Fmoc-arginine-glycine-aspartate (Fmoc-RGD) short peptides containing MNP in biocompatible and biodegradable hydrogels with physicochemical, mechanical and biological methods [223]. This magnetic short peptide hydrogel shows enhanced mechanical properties [224] and demonstrates faster and better self-healing properties and stability, with potential as a 3D scaffold for cell growth. Moreover, the enhanced mechanical stability and good injectability exhibited in the magnetic hydrogel with good biocompatibility and non-toxicity make it an ideal biomaterial for in vivo biomedical applications in minimally invasive surgery.

Traumatic hemorrhage is usually treated with compression hemostasis or vascular clamping. However, compression hemostasis carries the risk of vascular entrapment, which in turn leads to tissue necrosis in the corresponding blood supply area [225]. Some tissue engineering materials, such as hydrogels, sponges and nanofibers, are being used to staunch bleeding [226,227]. However, due to the lack of hemostatic activity and biosafety, most of the investigated materials struggle to be further translated by extension. Some short peptides exhibit good hemostatic effects by affecting different links in the coagulation process. The design strategy of peptide-based hemostatic materials consists of two aspects: 1. Mimicking key factors or enzymes in the coagulation process, such as various coagulation factors (factor VWF, factor VII) [228] and platelet receptor agonists (PAR1) [229] to activate platelets or promote thrombosis, thereby accelerating clotting. 2. A hydrogel system with a three-dimensional structure was constructed using self-assembling peptides to spontaneously form nanofibers under physiological conditions. A rapid hemostatic effect is demonstrated by flipping the wound and blocking blood flow. Peptide-based hemostatic materials are non-toxic, biodegradable, highly biocompatible with promising development prospects in the future biomedical field [230]. RGD peptide is a specific ligand for platelet membrane glycoprotein GPIIb/IIIa, which exhibits a propensity for platelet activity to adhere to traumatic bleeding sites. Zhang et al. produced an injectable hydrogel based on the physical-chemical interaction of gelatin, alginate and RG5 by introducing RG5 peptides into the lumen of salt-site nanotubes (HNTs) to achieve injectability and in situ gelation of hydrogels [231]. In this system, the nano-size function of nanotubes facilitates the adhesion of particles to red blood cells, which promotes cell aggregation and improves the hemostatic efficiency. The hydrogel itself can absorb a large amount of water to further improve the hemostatic effect. The injectable hydrogel exhibits superior hemostatic effect, and TGase can induce the hydrogelation of Ac–I3QGK-NH2 [232]. The peptide dimer is formed from the monomer by intermolecular ε-(Υ-glutamyl)lysine heteropeptide binding. The dimers rapidly self-assemble into flexible and entangled nanofibers, and the original Ac–I3QGK-NH2 nanoribbons and the nascent nanofibers co-hydrogelate. As an enzymogen which naturally circulates in the blood, it can be converted to Factor XIIIa (an active TGase) during the bleeding process. The peptide provides faster and more effective in vivo hemostasis compared to other hemostatic methods or materials. Moreover, the peptide exhibits low cytotoxicity and no induction of non-specific immune responses.

## 5 Conclusion and outlook

Self-assembled short peptides hold great promise in drug delivery applications, antibacterial, imaging, and as emulsion stabilizers. Many studies mentioned in this review suggest their enormous diagnostic and therapeutic effects. In addition, it has a better intelligent and diverse development prospect in drug delivery. However, challenges are still present that impede the use of self-assembled short peptides in clinical trials. The most significant problem is the lack of theoretical insight into the formation mechanisms of the self-assembled supramolecular nanostructures. For example, the precise control of the morphological changes must be further evaluated. As a result, self-assembled short peptide-based conjugates are designed and synthesized to predict or track the self-assembly process and nanostructures. Next, it is necessary to build smart-responsive short peptides materials to achieve a more efficient drug delivery system. Multiple responsive building blocks increase the drug amount on the target organ and decrease the side effects. Additionally, the problem of in vivo stability and bioavailability still needs to be addressed due to the sensitivity of peptides to proteases. Last but not least, the stability and toxicity assessment need attention and many studies in the clinical translation should be performed.

## Credit author statement

Yang Shihua: Conceptualization, Writing-Original Draft. Wang Mingge: Methodology, Software. Wang Tianye: Software. Sun Mengchi: Formal analysis. Huang Hanwei: Software. Shi Xianbao: Validation. Duan Shijie: Validation. Wu Ying: Visualization. Zhu Jiaming: Validation, Investigation. Liu Funan：Writing-Review & Editing, Supervision.

## Declaration of competing interest

The authors declare that they have no known competing financial interests or personal relationships that could have appeared to influence the work reported in this paper

## Data Availability

Data will be made available on request.
